# 3D Impedimetric Microfluidic Membrane-Mimic Cassette (IM3) with Interdigitated Electrodes for Fouling Analysis at Membrane Interfaces

**DOI:** 10.1021/acsami.5c26362

**Published:** 2026-04-21

**Authors:** Najamuddin Naveed Khaja, Sreerag Kaaliveetil, Niranjan Haridas Menon, Sushma Yadav, Chetan Prakash Sharma, Sagnik Basuray

**Affiliations:** † Department of Chemical and Materials Engineering, 5965 New Jersey Institute of Technology, Newark, New Jersey 07102, United States; ‡ Department of Biomedical Engineering, New Jersey Institute of Technology, Newark, New Jersey 07102, United States

**Keywords:** fouling, interdigitated microfluidic membrane-mimic cassette, electrochemical impedance spectroscopy, 3D electric field, polystyrene latex beads, cake layer

## Abstract

Membrane fouling remains a major challenge in water treatment, biomedical, and pharmaceutical fields, yet understanding its mechanisms at the membrane interface remains difficult. Herein, we report a 3D impedimetric microfluidic membrane-mimic (IM3) cassette that integrates a porous membrane between microfluidic channels for real-time fouling investigation via electrochemical impedance spectroscopy (EIS). System validation using fluorescein and KCl electrolytes demonstrated reproducible charge-transfer resistance (*R*_ct_) values and confirmed cassette robustness. Colloidal fouling studies using 800 nm polystyrene latex (PS) beads demonstrated a clear concentration-dependent behavior. High particle loading (10^5^ particles/mL) caused rapid, severe impedance increases, indicating extensive pore blockage and cake layer formation. Low loading (10^1^ particles/mL) showed minimal changes, suggesting negligible fouling, as confirmed by scanning electron microscopy. The distribution of relaxation times (DRT) analysis revealed a single dominant relaxation peak at 10^–2^ s that grew stronger as fouling severity increased, confirming that pore blockage raised interfacial resistance through a unified charge-transfer mechanism rather than caused diffusion-limited processes. A quantitative fouling model using EIS-measured *R*_ct_ changes captured temporal and concentration dependencies through an exponential growth framework. Maximum fouling extent (*F*_max_) increased significantly with particle concentration (30% at 10^1^ to 215% at 10^5^ particles/mL), demonstrating concentration-dependent pore blockage. The fouling rate constant (*k*) showed weak concentration dependence and plateaued at high loadings, indicating the limitation by available deposition sites rather than particle transport. These findings show that for PS beads, fouling is mainly governed by the extent of surface deposition (driven by concentration) rather than by the particle arrival rate (controlled by kinetics). The developed modular IM3 cassette enables customization of different membranes, channel geometries, and flow configurations. These results establish a quantitative framework linking EIS data to physically meaningful fouling phenomena, offering a versatile platform for the mechanistic investigation of dynamic membrane fouling behavior under varying particulate conditions.

## Graphical abstract

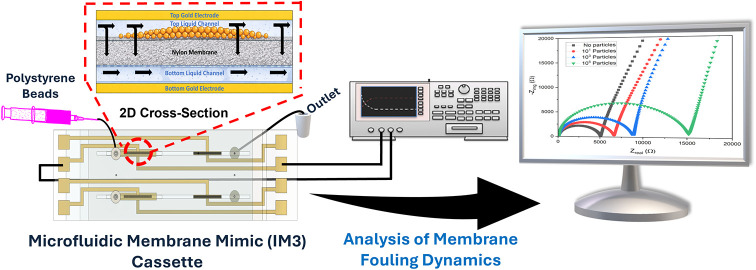

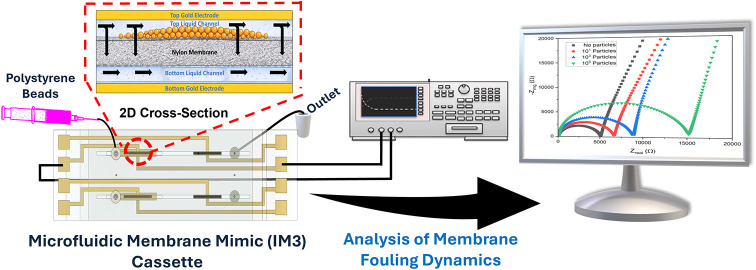

## 1 Introduction

Membrane technologies are crucial for supporting the global desalination industry and ensuring a freshwater supply in water-scarce regions.^1^ In the biomedical sector, membranes enable essential vital processes, including hemodialysis, plasma separation, and extracorporeal oxygenation.^2^ In pharmaceutical downstream processing, membranes are critical for sterile filtration of drug products, virus removal, and protein purification, where their high selectivity and scalability enable compliance with regulatory requirements.^3^ Additionally, in diagnostics and point-of-care applications, porous membranes provide both separation and sensing interfaces in lateral flow assays, lab-on-chip platforms, and microfluidic cartridges.^4^ Across these diverse applications, membranes offer advantages over conventional separation methods, including lower chemical consumption, reduced footprint, and compatibility with renewable-driven operations. Despite their versatility, the long-term performance of membranes is persistently limited by fouling.^5^

In recent decades, various strategies have been explored to reduce membrane fouling on ceramic and polymeric membrane surfaces. The unique properties of nanomaterials and their integration with state-of-the-art membranes were utilized to develop reactive membranes via different modification strategies, including surface coating, surface grafting, LBL assembly, and surface functionalization.^6–9^ However, membranes are confronted with the inevitable phenomenon of membrane fouling, which, over time, poses a significant obstacle to membrane performance.^10,11^

Traditional methods, such as measurement of pressure drop within the filtration, only respond to fouling formations.^12,13^ However, the early detection and prediction of fouling extent are challenging, particularly in industrial-scale membrane modules.^11^ To date, the cake layer formed on fouled membranes has been studied using ex situ techniques, such as membrane autopsy-based scanning electron microscopy (SEM) and other sophisticated microscopy methods (e.g., atomic force microscopy). Nevertheless, the interaction between foulant particles and fluid flow cannot be predicted solely from that. Therefore, novel techniques based on optical and nonoptical signal detection have been explored to characterize fouling during the process.^14,15^ Chen et al. used stimulated Raman scattering (SRS) microscopy to precisely detect 3D distributions of Bovine serum albumin (BSA) and polysaccharides fouling on a PVDF microfiltration membrane.^16^ Li et al. used optical coherence tomography (OCT) imaging to determine the in situ presence of silica nanoparticles and bentonite particles on an ultrafiltration membrane.^14^ Recently, Tanudjaja et al. combined OCT with a deep convolutional neural network (CNN) to accurately estimate biofouling thickness from membrane surface images.^11^

The integration of electrochemical impedance spectroscopy (EIS) to detect membrane fouling and monitor its growth has also been explored as a sensitive, nondestructive technique. Sim et al. used the EIS measurement to study the impact of silica, BSA, and their mixture on the surface of the RO membrane. They suggested that an EIS system connected to the spiral-wound module(s) could act as a “canary” cell to monitor membrane performance.^17^ After that, different groups explored integrating membrane fouling with EIS to determine real-time membrane performance across various types of advanced membranes, including single-walled/double-walled carbon nanotube-coated microfiltration membranes^18^ and ultrafiltration membranes.^19^ Although all previous studies focused on using solid electrode materials for EIS-based membrane fouling measurements, advances in materials science, membrane technology, and the development of electrochemical microfluidic devices can now enable the reduction in material and energy consumption.^20–23^ Therefore, we identified three key features that can enhance the synergistic combination of these developments. In this work, we hypothesize using a 3D interdigitated microfluidic device to (1) simulate membrane filtration using microfluidic channels, (2) study the interaction of polystyrene latex (PS) beads on the membrane surface using EIS, and (3) investigate the governing phenomena related to membrane fouling.

Herein, we present a nylon-membrane-integrated IM3 cassette for electrochemical characterization. The cassette incorporates a porous nylon membrane within a microchannel network, allowing direct interrogation of ionic transport and fouling under controlled hydrodynamic and chemical conditions. The IM3 cassette is enclosed by digitated microelectrodes on both the top and the bottom. This nonplanar structure, bounded by the microelectrodes, creates a 3D electric field that extends from the top to the bottom of the device, increasing sensitivity to membrane changes. 800 nm PS beads were selected as model foulants for a 450 nm pore-size nylon membrane to simulate the size-exclusion and deposition processes typical of practical filtration systems. PS beads are widely used in membrane fouling research because they provide a well-defined and reproducible colloidal model with controlled particle size, surface charge, and surface chemistry. Their use enables a systematic study of fundamental fouling mechanisms, such as particle deposition, surface coverage, and cake layer formation, while minimizing the complexity and variability encountered with real-world foulants, such as bacterial cells, colloids, and other submicron particles commonly encountered in water treatment and biomedical filtration.^18,24–29^ Their size, larger than the average membrane pore (450 nm), allows them to simulate surface deposition and pore-blocking processes that mimic the early stages of biofouling and particulate fouling in practical systems.^30^ The cassette was first validated using a fluorescence leak test to confirm a robust seal and then characterized electrochemically through impedance measurements at different KCl concentrations (1, 10, and 100 mM) to assess stability and repeatability. The fouling experiments demonstrate that the IM3 cassette is highly sensitive to concentration-dependent particulate fouling, with impedance spectra distinguishing between severe, moderate, and negligible obstruction regimes. The change in EIS spectra was further analyzed by using DRT and time-dependent impedance evolution via an exponential growth model. The results presented in this work demonstrate that the IM3 cassette can serve as a reliable and reproducible framework for probing fouling dynamics and transport phenomena. It underscores its broader potential as a flexible platform for diverse membrane-related applications.

## 2 Materials and Methods

### 2.1 Reagents and Instruments

Standard glass slides (product no.: 1301, Globe Scientific, Mahwah, NJ, USA) for assembling the cassette were obtained from Fisher Scientific. Hydrophilic nylon (NY) microfiltration membranes (product no. 1213776) with a pore size of 0.45 μm were obtained from MSE Supplies, Tucson, AZ, USA. Double-sided pressure-sensitive tape with thicknesses of 140 μm (ARcare 90106NB) was obtained from Adhesives Research, Glen Rock, PA, USA, and used to fabricate the microchannel. The syringe pump, NE-1000, used to inject the electrolyte solution (KCl) into the microfluidic channel was obtained from New Era Pump Systems Inc., East Farmingdale, NY, USA. The 3 mL BD Luer-lock syringe used to contain the KCl solution was obtained from Fisher Scientific (Waltham, MA, USA). A Luer lock compatible with 1/16 in. ID tubing was obtained from Amazon. The 1/16 in. ID, 3/16 in. OD Tygon tubing (product no.: 6516T62), made of PVC plastic, was obtained from McMaster-Carr and used to connect the syringe to the microfluidic platform. The 4294A Precision Impedance Analyzer from Keysight Technologies was used for all of the EIS measurements. Potassium chloride (KCl), ACS grade, was acquired from British Drug Houses (BDH). The deionized (DI) water used in the experiments was obtained from a Milli-Q Direct 8 Water Purification System. Pure fluorescein was purchased from Thermo Fisher Scientific (Catalog no.: 119240250). Fluorescence imaging was performed using a Nikon Eclipse TS2 inverted microscope with a FITC filter set. For fluorescence microscopy, a 1 mM fluorescein solution was prepared by dissolving 3.32 mg of fluorescein in 10 mL of NaOH. PS beads (800 nm diameter, 1 × 10^12^ particles/mL, Sigma-Aldrich Inc., catalog no. LB8–1 ML) were purchased and used as model foulants in the membrane fouling experiments. An Agilent Cary 670 Fourier-transform infrared (FTIR) spectrometer equipped with a diamond ATR bench was used to record spectra over 400–4000 cm^–1^. The surface morphology of the membranes was examined by using a field-emission scanning electron microscope (FE-SEM, JEOL JSM-7900F).

### 2.2 Microfabrication of Gold Microelectrodes and the Schematic of the Device

The gold microelectrodes are fabricated according to the method described in Figure 1A.^31–33^ First, a thin layer of positive photoresist (AZ1512) was spin-coated onto the cleaned glass slide to define the electrode pattern. The coated substrate was then exposed to UV light through a photomask, transferring the designed electrode geometry to the resist. After development, the exposed regions of the photoresist were removed, leaving behind well-defined trenches for metal deposition. A metal stack consisting of 10 nm Ti (adhesion layer) and 100 nm Au was deposited by using electron-beam evaporation to form the conductive electrode features. Finally, a lift-off step in the solvent (acetone) removed the remaining photoresist, yielding clean, sharply defined gold electrodes suitable for electrochemical measurements in the IM3 cassette.

**Figure 1:**
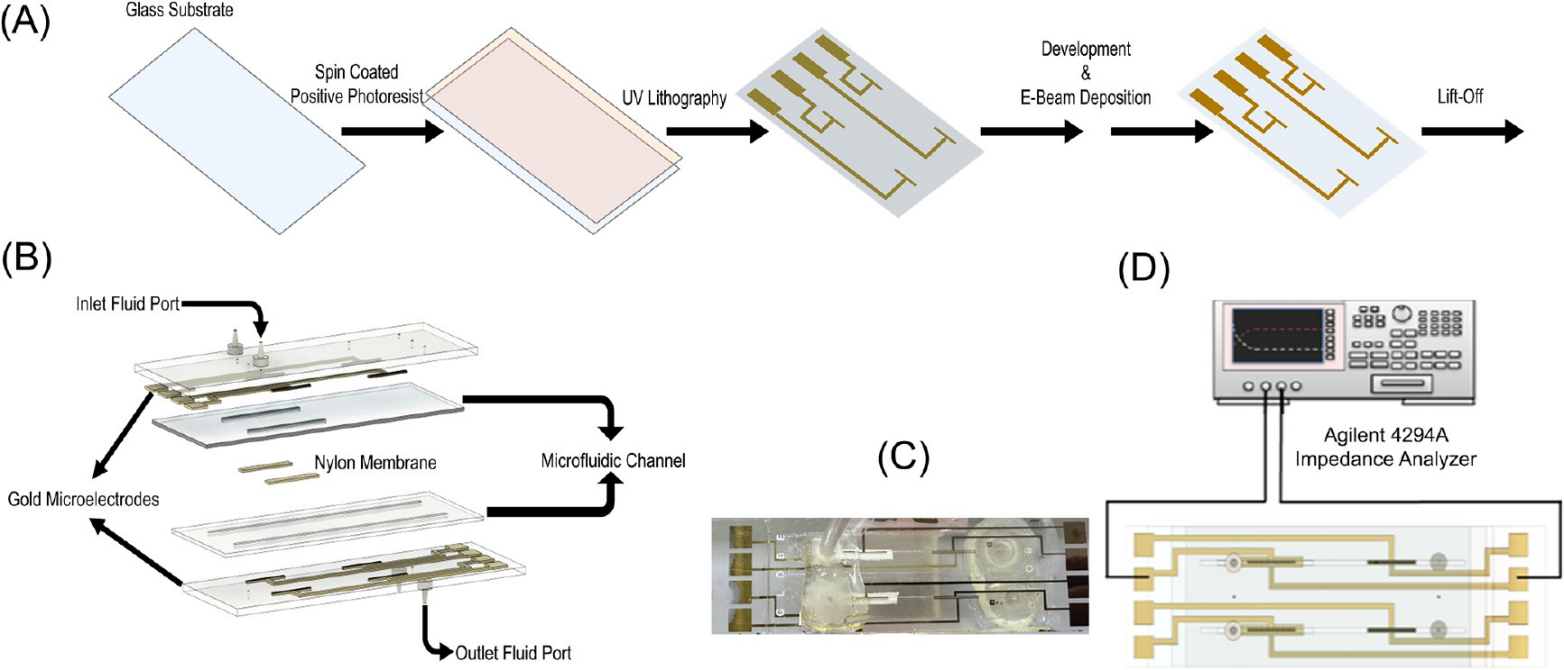
(A) Microfabrication procedure of the gold microelectrodes, (B) assembly of the IM3 cassette, (C) top view of the real IM3 cassette, and (D) electrode configurations of the IM3 cassette.

The assembly of the IM3 cassette is illustrated in Figure 1B, which shows the cassette assembled into five layers. The top layer consists of a glass substrate patterned with gold microelectrodes, which features an inlet port for introducing the electrolyte or test solution. The second layer is a microfluidic channel defined in double-sided adhesive tape that directs the injected solution toward the membrane interface. The third layer is a hydrophilic nylon membrane, positioned precisely within a slot cut into an additional adhesive layer. This configuration ensures that the membrane is sandwiched between the top and bottom microelectrodes and aligned with the first set of electrodes, as shown in Figure 1B, enabling direct monitoring of the transmembrane transport. The fourth layer is a bottom microfluidic channel bonded to a glass substrate containing gold microelectrodes. This bottom substrate features an outlet port at the channel end, allowing the electrolyte or test solution to exit the cassette. The top view of the real IM3 cassette is shown in Figure 1C.

The top electrode of the IM3 cassette was connected to the low terminal of the Agilent Impedance Analyzer. In contrast, the bottom electrode was connected to the high terminal, as shown in Figure 1D. The connection of electrodes to the high- or low-terminal impedance analyzer was not critical, as the EIS measurement reflects the response across the top and bottom electrodes of the membrane. The detailed experimental setup is explained in Section S1.

The liquid passed through the inlet microchannel, reached the membrane interface, and cross-flowed into the bottom microchannel (Figure S1B). From there, the flow exited the outlet port and entered the outlet reservoir. The appearance of the first drop at the outlet was defined as the starting point (0 min), at which impedance spectra were recorded over the frequency range of 40 Hz to 110 MHz using an oscillator (OSC) level of 500 mV. Subsequent measurements were collected every 15 min until the cassette reached a stable response. The acquired data were transferred to the computer system connected to the impedance analyzer via the instrument’s data acquisition software.

### 2.3 Detailed Fabrication Procedure of the Microfluidic Membrane Device

The detailed fabrication process for the microfluidic membrane device is shown in Figure 2. First, two holes, one for the fluid inlet and one for the liquid outlet, are drilled on the top and bottom microelectrode glass slides by using a drilling machine with a 1 mm diameter drill bit. Before assembling the device, the top and bottom slides (Figure 2A) are cleaned with acetone and IPA and dried at room temperature. A 140 μm-thick double-sided pressure-sensitive tape was used as the intermediate adhesive layer, as shown in Figure 2B, which also serves as the inlet and outlet microfluidic fluid channels. A 140 μm-thick double-sided pressure-sensitive tape was used as the intermediate adhesive layer and simultaneously defined the inlet and outlet microfluidic channels (Figure 2B). The channels were patterned using a Cricut Maker (Figure 2C); the inlet top channel was 22.86 mm long and 0.50 mm wide, while the bottom outlet channel was 43.18 mm long and 0.50 mm wide. The nylon membrane with a pore size of 0.45 μm was also cut using the same Cricut Maker, with dimensions of 12.70 mm in length and 2.38 mm in width. The fluid microchannels and membrane after a precise cut are shown in Figure 2D.

**Figure 2:**
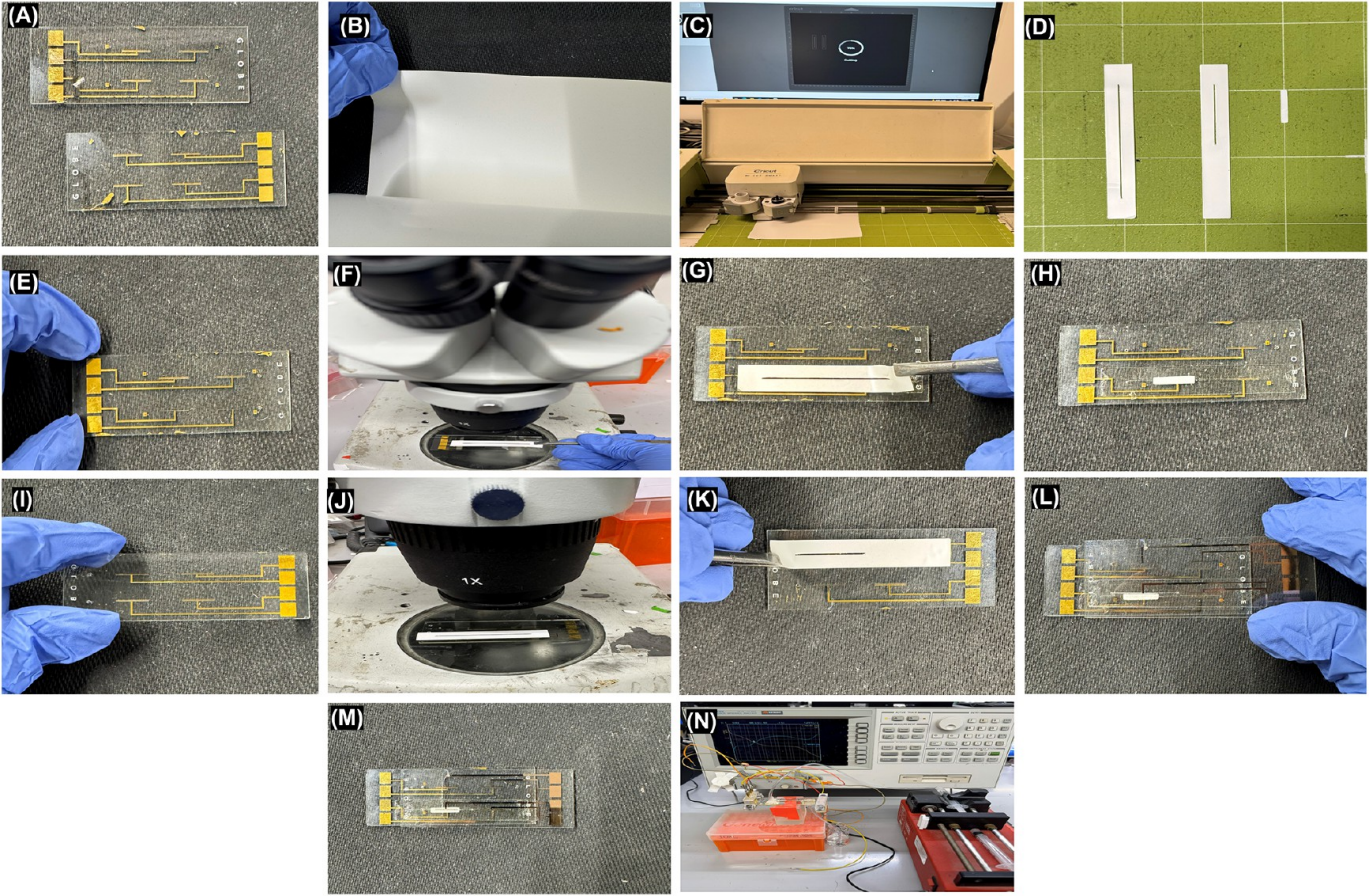
Detailed fabrication protocol of the device. (A) Bottom and top gold microelectrodes, (B–D) cutting of microchannels and membrane using Cricut Maker, (E–G) alignment of bottom microchannel on the bottom gold microelectrode, (H) placement of membrane on the bottom microchannel, (I–K) alignment of top microchannel on the top gold microelectrode, (L, M) alignment of microelectrodes and final IM3 cassette, and (N) electrochemical measurement setup of the device.

To assemble the IM3 cassette, first, the bottom outlet channel was aligned on the bottom microelectrode glass slide using a microscope, ensuring that the microelectrodes are within the channel geometry, as shown in Figure 2(E–G). After this, the top layer of the tape is peeled off, and the membrane is aligned with the channel, ensuring that all of the fluid contacts the membrane, as shown in Figure 2H. Then, to align the top electrode with the inlet microchannel, the inlet channel was precisely positioned over the electrode so that the inlet fluid flowed only across the membrane surface (Figure 2I,2J). After removal of the top tape layer (Figure 2K), the top glass slide was flipped and aligned with the bottom glass slide, ensuring alignment of the inlet–outlet microchannels and both microelectrode layers (Figure 2L).

The IM3 cassette features a five-layer architecture, where the membrane is sandwiched between the inlet and outlet microchannels and the top and bottom microelectrodes. The two glass slides are pressed gently to remove air bubbles, and the final device is shown in Figure 2M. Finally, the experimental setup is shown in Figure 2N. This modular assembly of the IM3 cassette enables customization for different membranes, channel geometries, and flow directions, accommodating a wide range of applications.

### 2.4 Bead Fouling Protocol

The fouling experiments were performed sequentially under controlled conditions. First, the IM3 cassette was primed with KCl electrolyte (10 mM), and impedance spectra were recorded at 15 min intervals until the system reached stabilization, defined as the overlap of three consecutive Nyquist plots. After stable baseline spectra were established, PS beads suspended in KCl electrolyte were introduced into the cassette under continuous flow. Impedance measurements were then recorded over time to monitor the progression of fouling. Experiments were conducted at multiple bead loadings to assess the concentration-dependent fouling behavior. Details of bead suspension preparation are provided in Section S2 and Table S1.

## 3 Results and Discussion

### 3.1 Validation of IM3 Cassette Integrity by Fluorescence Microscopy

The integrity of the IM3 cassette was verified using fluorescence microscopy with a 1 mM solution of fluorescein, as shown in Figure 3. Before introducing the dye, a background image was acquired for any autofluorescence from the membrane or microchannels. The fluorescein solution was introduced at a steady flow rate and imaged using an FITC filter. As the solution advanced from the inlet reservoir into the microchannel, the fluorescent signal appeared immediately and remained confined to the channel footprint. As shown in Figure 3A, the fluorescein solution was first filled into the inlet reservoir. When the liquid reached the membrane interface, a fluorescent signal became visible at the entrance (Figure 3B). Subsequently, the solution advanced steadily through the channel, as shown in panels of Figure 3C–F, illustrating the progressive filling of the entire microchannel. At all stages, the fluorescence remained confined to the channel boundaries with no detectable leakage into the surrounding regions, confirming the structural integrity of the IM3 cassette. These results confirm that the IM3 cassette was leak-free and suitable for subsequent electrochemical measurements.

**Figure 3:**
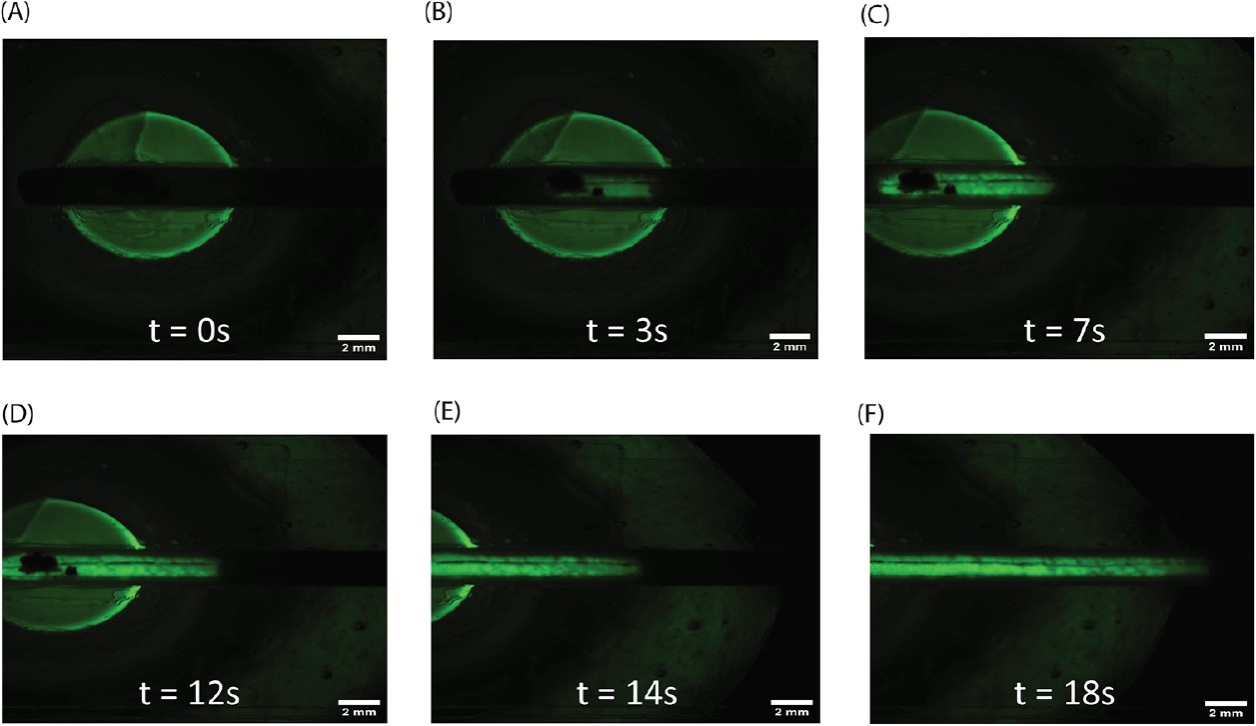
Fluorescence microscopy images of the IM3 cassette during leak testing with fluorescein (1 mM in NaOH, with time). (A) Fluorescent solution filling the inlet reservoir, (B) dye reaching the membrane interface, and (C–F) progressive filling of the microchannel until complete channel occupancy. Fluorescence remains confined within the channel boundaries throughout, confirming steady flow and absence of leakage into surrounding regions.

### 3.2 Effect of Electrolyte Concentration on Impedance Response

After the IM3 cassette was assembled and the EIS setup was connected, a syringe filled with 1 mM KCl was mounted on the syringe pump, and the electrolyte was delivered at a constant flow rate of 3 μL/min. The nylon membrane requires time to hydrate and fully establish consistent ionic pathways across its pores. At early time points, incomplete wetting may lead to higher charge-transfer resistance and greater transport variability, which gradually stabilize as the membrane becomes uniformly wet with the electrolyte. Therefore, the appearance of the first drop at the outlet port was taken as the starting point (0 min). The impedance spectrum was then recorded at 15 min intervals. System stabilization was confirmed by the overlap of three consecutive Nyquist plots (Figure 4A), indicating that the electrolyte–membrane interface had reached steady state. In the IM3 cassette, the Nyquist spectra reveal a semicircle at intermediate frequencies, associated with charge-transfer resistance and electrical double-layer capacitance, followed by a diffusion-controlled tail at low frequencies, characteristic of Warburg impedance.^34–36^ This spectral profile confirms that the cassette captures interfacial electrochemical processes and membrane-mediated ionic transport, with the relative contributions strongly dependent on the electrolyte concentration.

**Figure 4:**
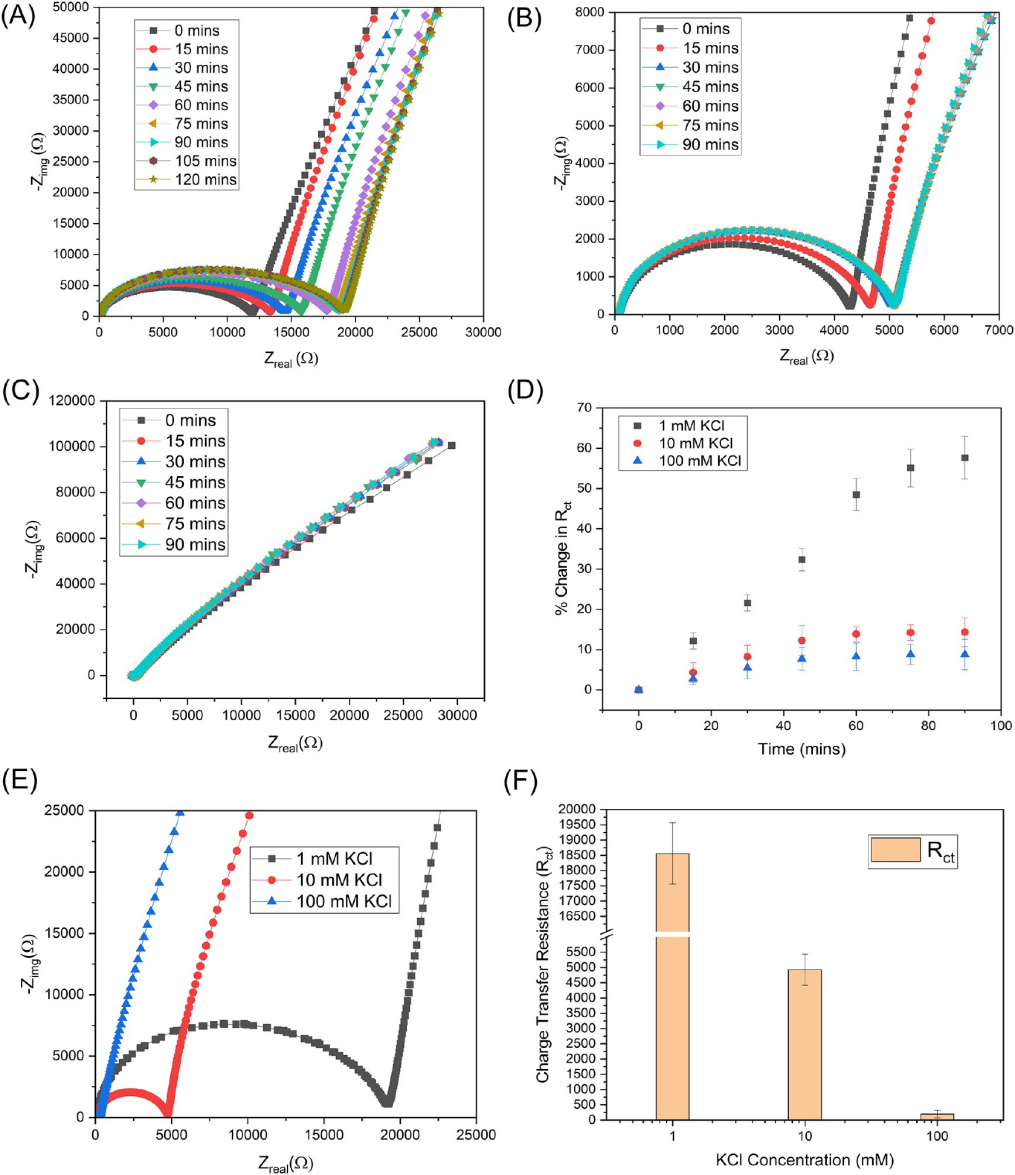
Electrochemical characterization of the IM3 cassette with KCl electrolytes at different concentrations. (A) Nyquist plots for 1 mM KCl recorded over time until stabilization, (B) Nyquist plots for 10 mM KCl, (C) Nyquist plots for 100 mM KCl, (D) corresponding % change in *R*_ct_ over time for stabilization, (E) Nyquist plots recorded for 1, 10, and 100 mM KCl after stabilization, showing the expected decrease in impedance with increasing ionic strength, and (F) *R*_ct_ was extracted from an equivalent circuit fitting as a function of KCl concentration, confirming the concentration-dependent reduction in resistance.

Once stability was established with 1 mM KCl, the flow was stopped, and the data were saved for analysis. A newly assembled IM3 cassette was prepared and filled with 10 mM KCl. The same procedure was performed under identical flow conditions, with impedance spectra collected every 15 min until three successive Nyquist plots overlapped. Figure 4B shows the Nyquist plots obtained at consecutive time points for 10 mM KCl. For the 100 mM KCl experiments, a separate, newly assembled IM3 cassette was used and tested under the same protocol. As shown in Figure 4C, the Nyquist plots again converged as the system approached equilibrium.

The impedance data were analyzed in ZView using a modified Randles circuit model (as described in Section S3 and Figure S2), and the charge-transfer resistance (*R*_ct_) was extracted at each time point. The resulting % change in *R*_ct_ values over time for 1-, 10-, and 100- mM KCl solutions is shown in Figure 4D. At a lower ionic strength (1 mM KCl), the system exhibited a relatively significant and gradual increase in *R*_ct_, reaching nearly 60% after 90 min, before approaching a steady value. This behavior indicates a more extended equilibration period, likely due to slower ion redistribution and slower double-layer stabilization under dilute electrolyte conditions. In contrast, for 10 and 100 mM KCl, the % change in *R*_ct_ remained below 15% throughout the measurement window, with the values converging after approximately 60–90 min. The rapid stabilization at higher concentrations is attributed to improved ionic conductivity and faster charging of the electrical double layer at the electrode–electrolyte interface. Collectively, the convergence of % change in *R*_ct_ values over time and their low drift beyond 90 min provides a quantitative confirmation of the system’s stability and reproducibility under continuous flow operation.

The stabilization behavior of the IM3 cassette showed a clear dependence on the electrolyte concentration. At low ionic strength (1 mM KCl), the limited availability of charge carriers slowed both charge transfer and diffusion, resulting in higher impedance values and longer stabilization times. In contrast, at higher concentrations (10 and 100 mM KCl), the greater ion abundance facilitated ionic conduction, resulting in faster equilibration and reduced spectral drift. These observations indicate that equilibrium is reached more quickly under conditions of higher ionic conductivity, whereas dilute electrolytes are associated with slower membrane hydration dynamics.

As shown in Figure 4E, the electrochemical response of the IM3 cassette was systematically evaluated at KCl concentrations of 1, 10, and 100 mM to examine the effect of ionic strength on charge-transfer resistance. The Nyquist plots show a clear dependence of the impedance on the electrolyte concentration. At 1 mM KCl, the impedance spectrum shows the largest semicircular arc, corresponding to the highest *R*_ct_ and limited ionic mobility across the membrane interface. As the concentration increased from 10 to 100 mM, the semicircle diameter decreased, reflecting enhanced ionic conductivity and reduced charge-transfer resistance. Figure 4F presents the fitted *R*_ct_ values extracted from an equivalent circuit analysis. A sharp decrease in *R*_ct_ was observed as the KCl concentration increased from 1 to 10 mM, followed by a minor reduction at 100 mM. This trend aligns with classical electrochemical theory, in which higher ionic strength increases the charge-carrier density, compresses the electrical double layer, and lowers the energy barrier for charge transfer across the electrode–electrolyte interface.^32,37^

Impedance measurements were performed under no-flow conditions to evaluate the IM3 cassette’s stability further. After the system was initially stabilized with 1 mM KCl for 120 min, the syringe pump was stopped, and the impedance spectra were collected every 15 min to monitor potential drift in the absence of flow, as has been observed by our group in other membrane-based microfluidic sensors for gas analysis.^38^ As shown in Figure S3A, the Nyquist plots recorded between 0 and 60 min nearly overlapped, displaying no appreciable change in the semicircle diameter. This behavior indicates that the electrolyte–membrane interface remains electrochemically stable even when the flow is halted after the initial stabilization period. The quantitative analysis of *R*_ct_ further supports the stability. As illustrated in Figure S3B, the *R*_ct_ change remained within 3% over the entire 60 min period, signifying minimal interfacial drift. These findings confirm that the IM3 cassette retains consistent electrochemical characteristics under dynamic and static conditions, highlighting the robustness and integrity of the assembled multilayer interface.

These findings demonstrate that the IM3 cassette is highly sensitive to variations in electrolyte concentration and can reproducibly capture concentration-dependent impedance responses. The strong correlation between ionic strength and *R*_ct_ further validates the IM3 architecture as a reliable and quantitative platform for probing the interfacial electrochemical behavior and ionic transport processes relevant to membrane-based systems.

### 3.3 Concentration-Dependent Fouling Studies in the IM3 Cassette

PS beads (10^5^, 10^3^, and 10^1^ particles/mL) were introduced into the IM3 cassette under continuous flow to intentionally foul the nylon membrane and evaluate its electrochemical response during pore blockage. The baseline Nyquist spectrum in 10 mM KCl, recorded after system stabilization (after 90 min), represents the clean membrane condition. Upon introducing the bead suspension, the Nyquist plots exhibited a progressive rightward shift over time, accompanied by an increase in the semicircle diameter. The detailed effects of bead deposition are explained in Section S6.

To confirm that these impedance changes originated from interfacial fouling rather than alterations in bulk electrolyte properties, the conductivities of pure 10 mM KCl solution and 10 mM KCl solution containing PS beads were measured by using a conductivity meter. In both cases, the conductivity was approximately 161 μS/cm, confirming that the introduction of beads did not significantly alter the electrolyte’s ionic strength or bulk conductivity. Therefore, the observed increase in charge-transfer resistance can be attributed primarily to fouling at the membrane interface rather than to any intrinsic change in solution conductivity.

FTIR studies clearly showed that the PS beads were deposited on the membrane surface (Figure S6). The changes in the FTIR spectra before and after PS bead exposure are also described in Section S4. This demonstrates that PS deposition does not chemically alter the nylon structure; instead, it leads to surface coverage and attenuation of the nylon’s functional groups, consistent with the formation of a physical fouling layer. The coexistence of both nylon and PS spectral features indicates that the PS beads accumulate to form a cake-like layer over the nylon membrane.

The scanning electron microscopy (SEM) was used to visually assess membrane surface morphology before and after bead fouling, as shown in Figure 5. The pristine nylon membrane (Figure 5A) exhibits a clean, porous surface with well-defined pore openings and no particulate deposition. Upon exposure to a low bead concentration (10^1^particles/mL, Figure 5B), only low beads are observed on the membrane surface. At intermediate concentration (10^3^ particles/mL, Figure 5C), bead coverage becomes more pronounced, with clusters forming on the membrane surface. At the highest concentration (10^5^ particles/mL, Figure 5D), the membrane surface is densely covered with beads, forming a surface-associated fouling layer. SEM observations indicate that beads predominantly accumulate on the membrane surface, leading to pore-mouth obstruction and reduced effective pore accessibility. To further evaluate whether beads penetrate the membrane pores, cross-sectional SEM imaging was performed for the fouled membrane at 10^5^ particles/mL (as described in Section S5 and Figure S7). The cross-sectional analysis confirms that beads predominantly accumulate at the membrane surface and at pore entrances without evidence of deep-pore intrusion. This progressive increase in surface coverage with particle concentration is directly reflected in the electrochemical impedance response, confirming that higher bead loadings promote increased physical blockage at the membrane interface and more severe fouling of the nylon membrane. As surface coverage and pore-mouth constriction increase, ionic transport pathways across the membrane–electrode system become more tortuous and restricted, leading to an increase in *R*_ct._

**Figure 5:**
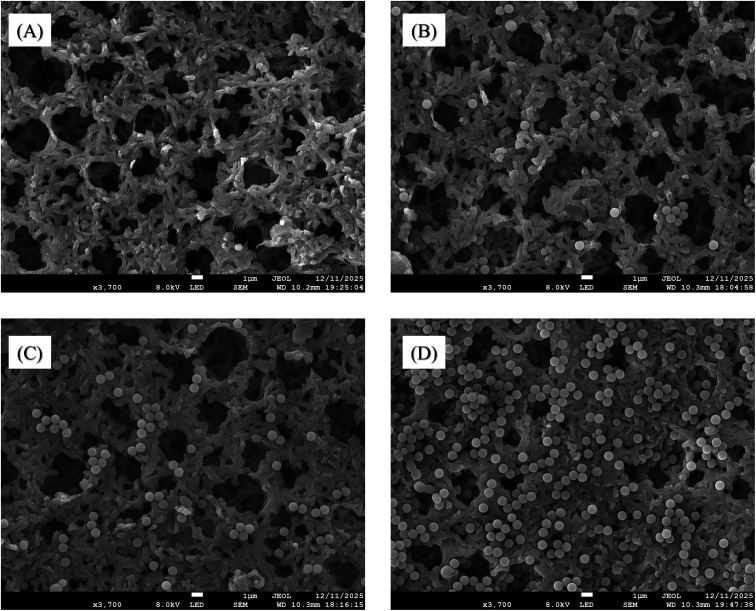
SEM images of (A) clean nylon membrane, (B) 10^1^ particles/mL fouled membrane, (C) 10^3^ particles/mL fouled membrane, and (D) 10^5^ particles/mL fouled membrane. The images reveal an apparent increase in bead deposition and surface coverage as particle concentration increases.

Figure 6A presents the Nyquist plots of the IM3 cassette recorded under varying concentrations of PS beads (10^1^, 10^3^, and 10^5^ particles/mL) suspended in 10 mM KCl, compared to the baseline (no particles). The clean membrane condition (black curve) shows the smallest semicircle, indicating minimal charge-transfer resistance and unhindered ionic transport across the membrane. As particle concentration increases, the Nyquist plots shift to the right, and the semicircle diameter increases, indicating an increase in charge-transfer resistance (*R*_ct_). At low bead loading (10^1^ particles/mL, Figure 6A-red curve), only a slight rightward shift is observed, corresponding to minor surface deposition and negligible pore obstruction. The intermediate concentration (10^3^ particles/mL, Figure 6A-blue curve) exhibits a more pronounced semicircular expansion, indicating a partial pore blockage and moderate fouling. In contrast, at the highest concentration (10^5^ particles/mL, Figure 6A-green curve), a substantial increase in both real and imaginary impedance components is evident, reflecting severe pore clogging and restricted ion transport through the membrane.

**Figure 6:**
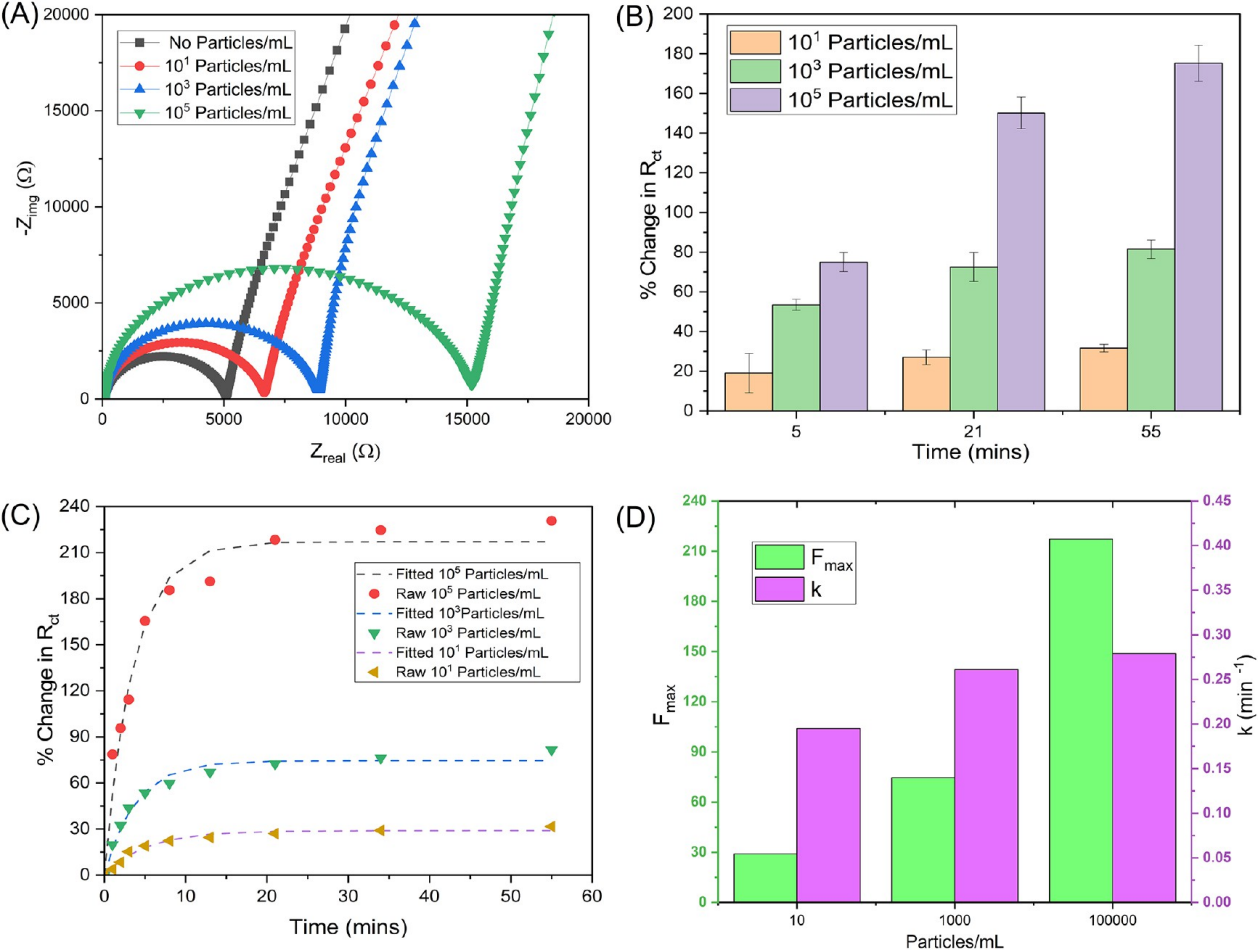
Fouling characterization of the IM3 cassette using 800 nm PS beads suspended in 10 mM KCl. (A) Nyquist plots at 55 min show the IM3 cassette’s impedance response after introducing bead suspensions of varying concentrations (no particles, 10^1^, 10^3^, and 10^5^ particles/mL) in 10 mM KCl electrolyte, (B) percentage change in charge-transfer resistance (%*R*_ct_) at 5, 21, and 55 min for three independent runs at each concentration, (C) comparison of experimental (%*R*_ct_) data (symbols) and nonlinear exponential fits (dashed lines) describing time-dependent fouling behavior for 10^1^, 10^3^, and 10^5^ particle concentrations, and (D) variation of fitted parameters, maximum fouling extent (*F*_max_, left axis), and fouling rate constant (*k*, right axis) as a function of particle concentration.

This systematic increase in impedance with particle loading confirms the concentration-dependent fouling behavior of the IM3 cassette. Since the bulk conductivity of the 10 mM KCl solution remained constant (∼161 μS/cm) with and without beads, the observed impedance increase arises primarily from interfacial phenomena rather than changes in solution conductivity. Thus, the FTIR, SEM, and EIS results together confirm that the fouling mechanism is dominated by surface deposition and cake layer buildup rather than chemical modification of the membrane material.

To ensure reproducibility, fouling experiments were conducted in triplicate for each bead concentration (10^1^, 10^3^, and 10^5^ particles/mL), and the results are summarized in Figure 6B as the percentage change in charge-transfer resistance (%*R*_ct_) over time with error bars representing the standard deviation. Across all concentrations, the %*R*_ct_ increased with exposure time, reflecting the progressive buildup of beads on the membrane surface and the corresponding rise in charge-transfer resistance. At 5 min, the increase in %*R*_ct_ was relatively modest for all concentrations, indicating the early stage of bead adsorption with limited pore interaction. By 21 min, a clear concentration-dependent trend emerged, with higher bead loadings producing significantly higher %*R*_ct_ values. The highest concentration (10^5^ particles/mL) showed the most significant increase, consistent with rapid pore blockage and interfacial accumulation. In contrast, the 10^3^ particles/mL showed a moderate increase in resistance, and the 10^1^ particles/mL remained comparatively stable. At 55 min, this trend became more pronounced, confirming that the extent of fouling scales with both time and particle concentration. The small standard deviations across all runs demonstrate the excellent repeatability and stability of the IM3 cassette measurements.

To quantitatively evaluate the fouling behavior observed in the IM3 cassette, the percentage change in charge-transfer resistance, *F*(*t*, *C*), was defined as the fouling index and was used as the key variable of interest. It represents the normalized increase in *R*_ct_ with time for a given bead concentration *C*, as expressed in eq 1

1 $$F\left(t,C\right)=\frac{R_{\mathrm{ct}}\left(t,C\right)-R_{\mathrm{ct},0}\left(C\right)}{R_{\mathrm{ct},0}\left(C\right)}\times 100$$

where *R*_ct_(*t*, *C*) is the charge-transfer resistance at time t and concentration *C*, and *R*_ct,0_(*C*) is the initial (baseline) resistance measured immediately after stabilization, corresponding to a clean membrane. This normalization allows a direct comparison of fouling dynamics across different particle concentrations, independent of their absolute baseline resistance values.

To model the time-dependent fouling behavior, the experimental data were fitted by using an exponential growth function, as shown in eq 2

2 $$F\left(t,C\right)=F_{\max}\left(C\right)\times \left(1-e^{-k\left(C\right)t}\right)$$

Here, *F*_max_(*C*) represents the maximum attainable fouling (asymptotic limit of *F*(*t*, *C*) for a given concentration), and *k*(*C*) is the apparent fouling rate constant that characterizes how quickly resistance approaches this limit. Because both *F*_max_ and *k* can depend on particle concentration, they were expressed as

3 $$F_{\max}\left(C\right)={\mathrm{AC}}^{m}\;\mathrm{and}\;k\left(C\right)=k_{0}C^{n}$$

where *A* and *k*_0_ are the fitting constants, and *m* and *n* describe the sensitivity of the maximum fouling extent and fouling rate, respectively, to the particle concentration. Combining these relationships gives the final concentration-dependent model

4 $$F\left(t,C\right)={\mathrm{AC}}^{m}\times \left(1-e^{-k_{0}C^{n}t}\right)$$

The fitting was performed using the nonlinear and linear curve-fitting tools in OriginPro, as described in Figures S10, S11, and S12. The fitting parameters were extracted for each concentration (10^1^, 10^3^, and 10^5^ particles/mL). The concentration- and time-dependent fouling were successfully fitted to the proposed exponential model, yielding the following empirical relationship

5 $$F\left(t,C\right)=17.170\times C^{0.218}\times \left(1-e^{-0.185C^{0.0389}t}\right)$$

Figure 6C compares the experimental %*R*_ct_ data with the corresponding nonlinear exponential fits for PS bead suspensions containing 10^1^, 10^3^, and 10^5^ particles/mL, introduced into the IM3 cassette under continuous flow. The raw data (symbols) represent the measured percentage increase in charge-transfer resistance over time, and the dashed lines denote the fitted curves derived from the proposed exponential fouling model.

At the highest particle loading (10^5^ particles/mL), a rapid rise in %*R*_ct_ is observed within the first few minutes, followed by a gradual approach to a steady plateau near 210%, indicating extensive pore blockage and the formation of a dense fouling layer. For the intermediate concentration (10^3^ particles/mL), the increase in %*R*_ct_ is slower and levels off at around 80%, suggesting partial pore obstruction and a thinner deposit layer. The system shows a smoother transition to a steady state, consistent with a moderate fouling rate and limited particle accumulation. At the lowest particle concentration (10^1^ particles/mL), the %*R*_ct_ remains below 40% even after 55 min, reflecting minimal fouling through the membrane.

The fitted exponential curves show excellent agreement with the experimental trends across all concentrations, validating the model’s ability to capture both the rate and extent of fouling. The results clearly demonstrate concentration-dependent fouling kinetics: higher bead loadings yield greater total resistance buildup (higher *F*_max_), confirming that the IM3 cassette can sensitively resolve the dynamic evolution of interfacial blockage under varying particulate conditions. Figure 6D illustrates how *F*_max_ and *k* vary with the particle concentration. *F*_max_ increases strongly with concentration, rising from ∼30% at 10^1^ particles/mL to ∼75% at 10^3^ and ∼215% at 10^5^ particles/mL. This monotonic rise indicates that higher bead loadings produce a larger overall increase in charge-transfer resistance, consistent with extensive fouling. In contrast, the fitted rate constant *k* also increased with concentration but began to plateau at the highest loading (10^5^ particles/mL), suggesting that while more particles accelerate fouling initially, surface saturation limits the rate of further deposition at later times. This indicates that the extent of fouling is concentration-controlled. In comparison, the kinetics are primarily governed by the local attachment of beads at the interface and the progressive reduction of available sites. This interpretation aligns with the proposed model in eq 5, which yields a positive exponent for extent (*m* = ∼0.218) but a near-zero exponent for rate (*n* = ∼0.0389). This indicates that *F*_max_ scales with concentration, while *k* remains weakly dependent on the concentration. The fitted results showed that increasing particle concentration significantly increased *F*_max_, confirming that higher particle loading led to more complete pore coverage and greater charge-transfer resistance. In contrast, *k* exhibited only a weak dependence on concentration, suggesting that the kinetics of fouling are limited by the availability of active deposition sites rather than by particle transport.

Although this platform does not directly measure flux or transmembrane pressure, the extracted parameters provide practical insight into the membrane fouling behavior. The *k* reflects the rate of resistance buildup analogous to early stage flux decline, while the *F*_max_ corresponds to the ultimate severity of fouling, comparable to steady-state resistance in filtration systems.^39–46^ Together, these parameters help distinguish between fouling kinetics and the long-term fouling extent under different operating conditions.

These results show that PS bead fouling in the IM3 device is primarily due to surface deposition with membrane blocking mainly influenced by particle concentration rather than by changes in the apparent fouling rate constant under the tested conditions. While monodisperse PS beads were used to identify basic trends, real-world membrane fouling involves a balance between a concentration-driven mass flux and additional kinetic processes. Higher foulant concentration increases the surface coverage, pore constriction, and cake formation, aligning with the strong concentration dependence of *F*_max_ observed here. In contrast, real foulants such as proteins, natural organic matter, and microorganisms introduce adsorption–desorption, structural changes, intermolecular interactions, and biological growth, making fouling kinetics more dependent on surface chemistry, hydrodynamics, and foulant interactions.^47–49^ Therefore, the current results establish a baseline of concentration-driven fouling, and the mechanistic separation between *F*_max_ and *k* offers a reference for understanding deviations in more complex chemical and biological systems.

The cumulative particle dose delivered to the membrane surface (as described in Section S7) was estimated using eq S3, *N*(*t*) = *CQt*/*A*, where *C* is the particle concentration (particles/mL), *Q* is the flow rate (mL/min), *t* is the time (min) of the bead deposition, and *A* is the membrane area (0.303 cm^2^). Figure 7A shows the evolution of the cumulative dose (*N*) with time for three different bead concentrations (10^1^, 10^3^, and 10^5^ particles/mL). The dose increased steadily over time and exhibited a clear dependence on particle concentration, spanning more than 4 orders of magnitude. The semilog plot shows that at a constant flow rate, higher particle concentrations deliver a significantly greater areal flux to the membrane, leading to faster particle accumulation per unit area. The curvature of the profiles at longer times suggests that the deposition rate gradually approaches a steady state, consistent with the formation of a surface fouling layer on the membrane.

**Figure 7:**
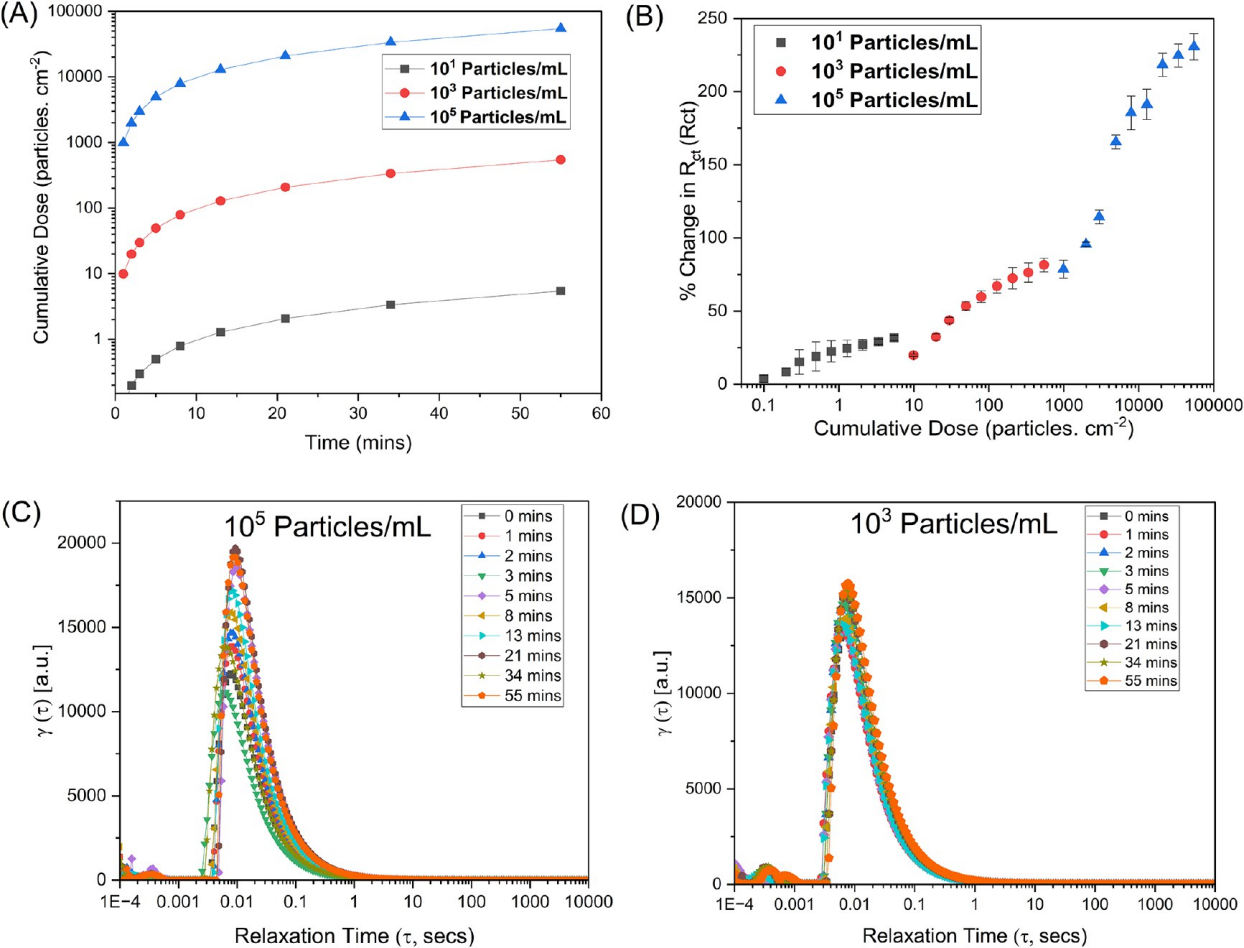
(A) Cumulative particle dose (*N*) with time at different bead concentrations. The semilog representation shows that higher particle loadings deliver significantly greater particle flux to the membrane interface, leading to faster foulant accumulation. (B) Relationship between cumulative particle dose (*N*) and percentage change in charge-transfer resistance (%*R*_ct_). The monotonic rise in *N* demonstrates that resistance growth directly tracks the number of particles arriving and adhering to the membrane surface, (C) distribution of relaxation times (DRT) analysis for 10^5^ particles/mL, and (D) DRT analysis for 10^3^ particles/mL.

To establish a quantitative relationship between fouling severity and cumulative particle deposition, the percentage change in charge-transfer resistance (%*R*_ct_) was plotted against the corresponding particle dose as shown in Figure 7B. The %*R*_ct_ increased monotonically with dose for all concentrations, confirming that *R*_ct_ growth directly correlates with the number of particles arriving at and adhering to the membrane surface. At low particle doses (10^1^ particles/mL), only a modest increase in the resistance was observed, indicating minimal pore blockage. Intermediate doses (10^3^ particles/mL) led to a stronger response, while high doses (10^5^ particles/mL) produced a sharp rise in %*R*_ct_, reflecting extensive pore occlusion and cake layer buildup. The semilog representation shows that resistance scales nonlinearly with the delivered dose, suggesting a transition from minimal to extensive pore blocking. Together, these results demonstrate that the IM3 cassette can effectively link electrochemical impedance data to physically meaningful fouling fluxes, providing a quantitative framework for interpreting dynamic membrane fouling behavior.

A distribution of relaxation times (DRT) analysis was also performed on the EIS spectra by using a regularization-based inverse Fourier transform method to gain deeper insight into the electrochemical processes contributing to impedance behavior during fouling. The DRT plots deconvolute the measured impedance into discrete relaxation processes characterized by their relaxation time constants (τ), providing a clearer view of interfacial phenomena that may overlap in the Nyquist representation.

Figure 7C presents the DRT spectra for PS bead concentrations of 10^5^ particles/mL under continuous flow conditions, and Figure 7D presents those for 10^3^ particles/mL. Each curve corresponds to a different fouling time, from baseline (clean membrane, 10 mM KCl) to 55 min after bead introduction. A single dominant peak centered at 10^–2^ s was observed for both concentrations, corresponding to the primary charge-transfer relaxation process at the electrode–electrolyte–membrane interface. During fouling, the peak intensity increased steadily over time, reflecting an increase in interfacial resistance as pores became progressively blocked by bead deposition. Notably, a single dominant relaxation peak remains throughout the entire fouling process. At the highest particle concentration (10^5^ particles/mL), this peak shifts slightly toward longer relaxation times as fouling progresses, whereas at lower concentrations, no significant shift is observed (Figure S13). This suggests that the same interfacial process changes over time rather than new relaxation mechanisms appearing. A gradual reduction in effective pore accessibility would affect the same interfacial relaxation process, leading to peak broadening rather than the emergence of additional relaxation modes. This behavior suggests that a single electrochemical mechanism, likely charge transfer across a partially obstructed interface, dominates the impedance response throughout the fouling process. In EIS, solution resistance typically appears at very short-time scales (<10^–4^ s), charge-transfer-related processes dominate intermediate time scales (10^–3^–10^–1^ s), and diffusion-controlled phenomena emerge at much longer time scales (>1 s). The dominant relaxation peak observed here near ∼10^–2^ s is therefore attributed to interfacial processes sensitive to ionic accessibility and pore-mouth constriction rather than to bulk diffusion or solution resistance effects.^50–52^

## 4 Conclusion

We developed and validated a 3D integrated microfluidic membrane-mimic (IM3) cassette that incorporates a porous membrane within a microfluidic platform for the EIS-based investigation of membrane fouling processes. Fluorescence leak testing confirmed cassette integrity, while baseline characterization with KCl electrolytes (1–100 mM) yielded reproducible Nyquist spectra and charge-transfer resistance values, establishing system robustness under controlled flow conditions.

The IM3 cassette successfully resolved membrane fouling dynamics using polystyrene latex beads as model foulants. Fouling experiments revealed concentration-dependent increases in charge-transfer resistance (*R*_ct_) corresponding to progressive pore blockage and surface deposition. The DRT analysis identified a single dominant relaxation peak at 10^–2^ s that intensified with increasing fouling severity, confirming a unified charge-transfer mechanism across the obstructed interface. Time-dependent impedance evolution was accurately captured by an exponential growth model, incorporating temporal and concentration dependencies. Fitted results showed that increasing particle concentration from 10^1^ to 10^5^ substantially enhanced maximum fouling extent (*F*_max_) from 30% to 215%, while the fouling rate constant (*k*) exhibited minimal concentration dependence (*n* ≈ 0.0389) and plateaued at high loadings. These findings show that fouling is mainly caused by surface deposition, a concentration-controlled process that is limited by the availability of deposition sites rather than by particle transport kinetics.

Since the presented results were obtained by sandwiching a microfiltration nylon membrane between 3D gold microinterdigitated electrodes of the same pattern at constant feed flow rates, further studies are needed to explore the effects of different electrode materials, various patterns,^22^ membranes, and feed solutions containing emerging pollutants of interest. Translating insights from this microfluidic-based study into practical applications will also depend on the choice of membranes, electrode materials, and patterns. Beyond fouling investigations, this platform can be easily adapted to study porous electrode–electrolyte interactions, ionic transport, membrane stability, drug dissolution, and diagnostic separations, providing broad utility across the environmental, biomedical, and pharmaceutical fields.

## Supplementary Material



## Notes

Detailed description of the experimental setup, schematic view of the experimental setup, and the 2D cross section of the IM3 cassette showing the crossflow pattern; preparation of polystyrene bead suspensions; table containing details related to preparation of polystyrene bead suspensions used for IM3 fouling experiments; details related to impedance data fitting using ZView with figure; figures related ZView fitting results, the circuit used with the obtained parameters and the raw vs the fitted results for the 1, 10, and 100 mM KCl; raw vs the fitted and circuit used with the obtained parameters for the no-flow 1 mM KCl; FTIR spectra analysis of the membrane pre- and post-PS bead exposure; cross-sectional SEM analysis of membrane fouling; effect of particle concentration on fouling progression in the IM3 cassette; fouling characterization of the IM3 cassette using 800 nm polystyrene latex beads suspended in 10 mM KCl; fitting results and parameters for fitting of 10^5^, 10^3^, and 10^1^ particles/mL at 55 min; nonlinear fit data that describes the *F*_max_ and *k* for the fit and the residual plots for the 10^5^, 10^3^, and 10^1^ particles/mL; linear fit data that describes the m and n for the final predicted model, the fit and the residual plots for m and n parameters, linear fit data that describes the m and n for the final predicted model; quantitative evolution of the dominant DRT relaxation peak at ∼10^–2^ s during fouling; and dose and flux calculations (PDF)
