# Supplementary material for: 3D Impedimetric Microfluidic Membrane-Mimic Cassette (IM3) with Interdigitated Electrodes for Fouling Analysis at Membrane Interfaces
Source: ACS Appl Mater Interfaces. 2026 Apr 21;18(17):25382–95. doi: 10.1021/acsami.5c26362 (PMC13154124; doi:10.1021/acsami.5c26362)
Supplement: Supplementary file 1 [file am5c26362_si_001.pdf]

Supporting Information

3D Impedimetric Microfluidic Membrane-Mimic Cassette (IM3) with Interdigitated Electrodes for Fouling Analysis at Membrane Interfaces

Najamuddin Naveed Khaja[a], Sreerag Kaaliveetil[a], Niranjan Haridas Menon[a], Sushma Yadav[a], Chetan Prakash Sharma[a]*, and Sagnik Basuray[a,b]*

[a]Department of Chemical and Materials Engineering, New Jersey Institute of Technology, Newark, New Jersey, 07102, United States of America

[b]Department of Biomedical Engineering, New Jersey Institute of Technology, Newark, New Jersey, 07102, United States of America

*Corresponding Authors:

Chetan Prakash Sharma: cs779@njit.edu and Sagnik Basuray: sbasuray@njit.edu

Keywords: Fouling, Microfluidic Membrane Mimic Cassette, Electrochemical Impedance Spectroscopy, 3D Electric Field, Polystyrene Latex Beads, Cake Layer

Section S1. Experimental setup

The experimental configuration is shown in **Figure S1A.** The electrolyte or test solution was loaded into a 3 mL syringe and mounted on an NE-1000 programmable single-channel syringe pump to deliver flow at controlled rates. The top electrode of the IM3 cassette was connected to the low terminal of the Agilent Impedance Analyzer, while the bottom electrode was connected to the high terminal. The connection of electrodes to the high- or low-terminal impedance analyzer was not critical, as the EIS measurement reflects the response across the top and bottom electrodes of the membrane. The syringe pump was set to the desired flow rate during operation, and the solution was introduced through the inlet port. The liquid passed through the inlet microchannel, reached the membrane interface, and cross-flowed into the bottom microchannel as shown in **Figure S1B**. From there, it exited the outlet port and entered the outlet reservoir. The appearance of the first drop at the outlet was defined as the starting point (0 min), at which impedance spectra were recorded over the frequency range of 40 Hz to 110 MHz using an oscillator (OSC) level of 500 mV. Subsequent measurements were collected every 15 min until the cassette reached a stable response. The acquired data were transferred to the computer system connected to the impedance analyzer via the instrument’s data acquisition software.

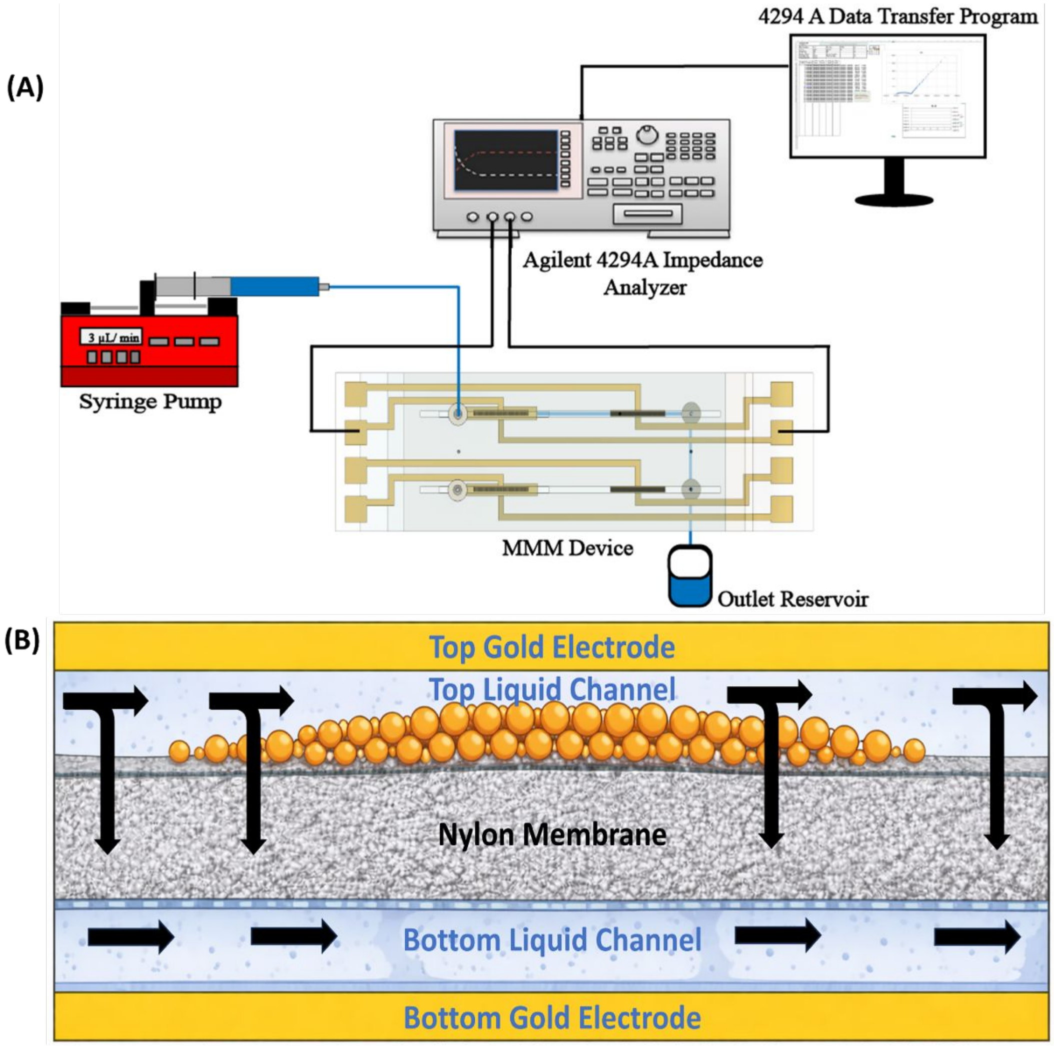

Figure S1: (A) Schematic view of the experimental setup. (B) The 2-D cross-section of the IM3 cassette showing the crossflow pattern.

Section S2. Preparation of polystyrene bead suspensions

Polystyrene latex beads (800 nm diameter, Sigma-Aldrich, catalog no. LB8-1ML) with a stock concentration of 3.57×10^{11} particles/mL were used as model foulants. Serial dilutions were performed in 10 mM KCl to obtain the desired bead concentrations for fouling experiments. The dilution procedure is summarized in Table S1. For each step, 10 µL of the previous suspension

was added to 990 µL of 10 mM KCl and vortex-mixed to ensure uniform dispersion. This serial dilution method produced bead concentrations spanning 10^5, 10^3, and 10^1 particles/mL, corresponding to the conditions tested in the IM3 cassette fouling experiments.

Table S1: Preparation of polystyrene bead suspensions used for IM3 fouling experiments.

Step	From	Particles added	Total Volume	Concentration (particles/mL)	Dilution Recipe
Stock	—	—	—	3.57×10^{11}	starting solution (stock)
1st	Stock	3.57×10^9	1 mL	3.57×10^9	10 µL stock + 990 µL KCl
2nd	1st	3.57×10^7	1 mL	3.57×10^7	10 µL of 1st dilution + 990 µL KCl
3rd	2nd	3.57×10^5	1 mL	3.57×10^5	10 µL of 2nd dilution + 990 µL KCl
4th	3rd	$3.57 \text{ x } 10^3$	1 mL	3.57×10^3	10 µL of 3rd dilution + 990 µL KCl
5th	4th	$3.57 \text{ x } 10^1$	1 mL	$3.57 \text{ x } 10^1$	10 µL of 4th dilution + 990 µL KCl

Section S3. Impedance data fitting using ZView

Unlike in a traditional Randles circuit, we have ignored the solution resistance ($R_{s)}$ due to the high ionic strength of the background electrolyte. We expect R_s to be substantial for lower-

ionic-strength background electrolytes; with this assumption, the Randles circuit is suitably modified in ZView® **(Figure S2A).** Electrochemical impedance spectroscopy (EIS) data were analyzed to extract the charge transfer resistance (R_{ct}) and associated circuit parameters.

As shown in **Figure S2B,** here, R_1 or R_{ct} represents the membrane resistance between the two electrodes. This effect is modelled as a membrane resistance, similar to TEER analysis.[1] CPE_1 models the double-layer capacitance and non-ideal charge accumulation at the electrode-electrolyte interface. CPE_2 represents interfacial polarization or diffusion-related behavior at the membrane–electrolyte interface, often accounting for surface-roughness and distributed-capacitance effects.

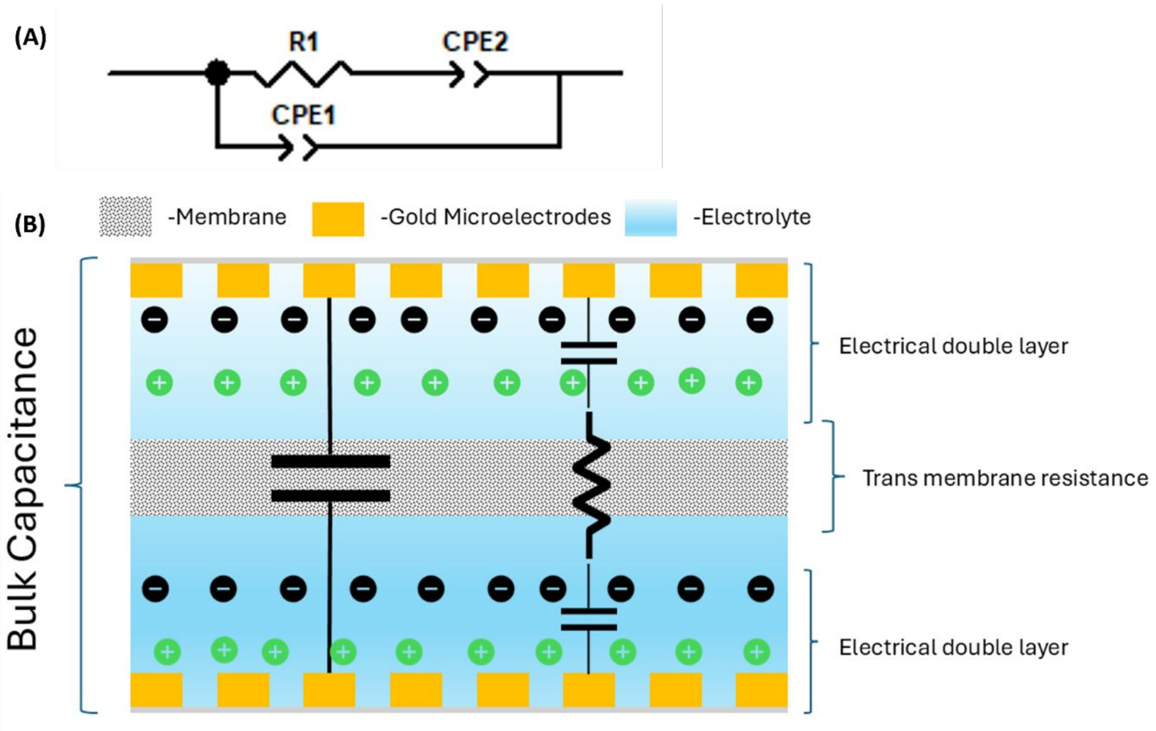

Figure S2: (A) Equivalent electrical circuit (RC). (B) Explanation of the equivalent electrical circuit model for the IM3 cassette.

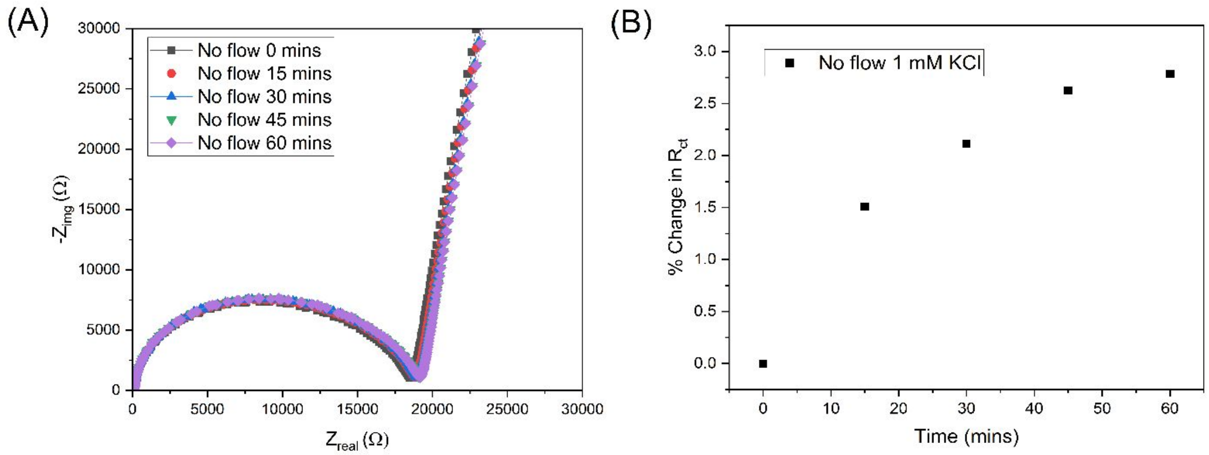

Figure S3: (A) Nyquist spectra of the IM3 cassette with 1 mM KCl collected between 0 and 60 min under no-flow conditions, showing no significant changes in impedance. (B) The corresponding % change in charge transfer resistance over time showed a stable response.

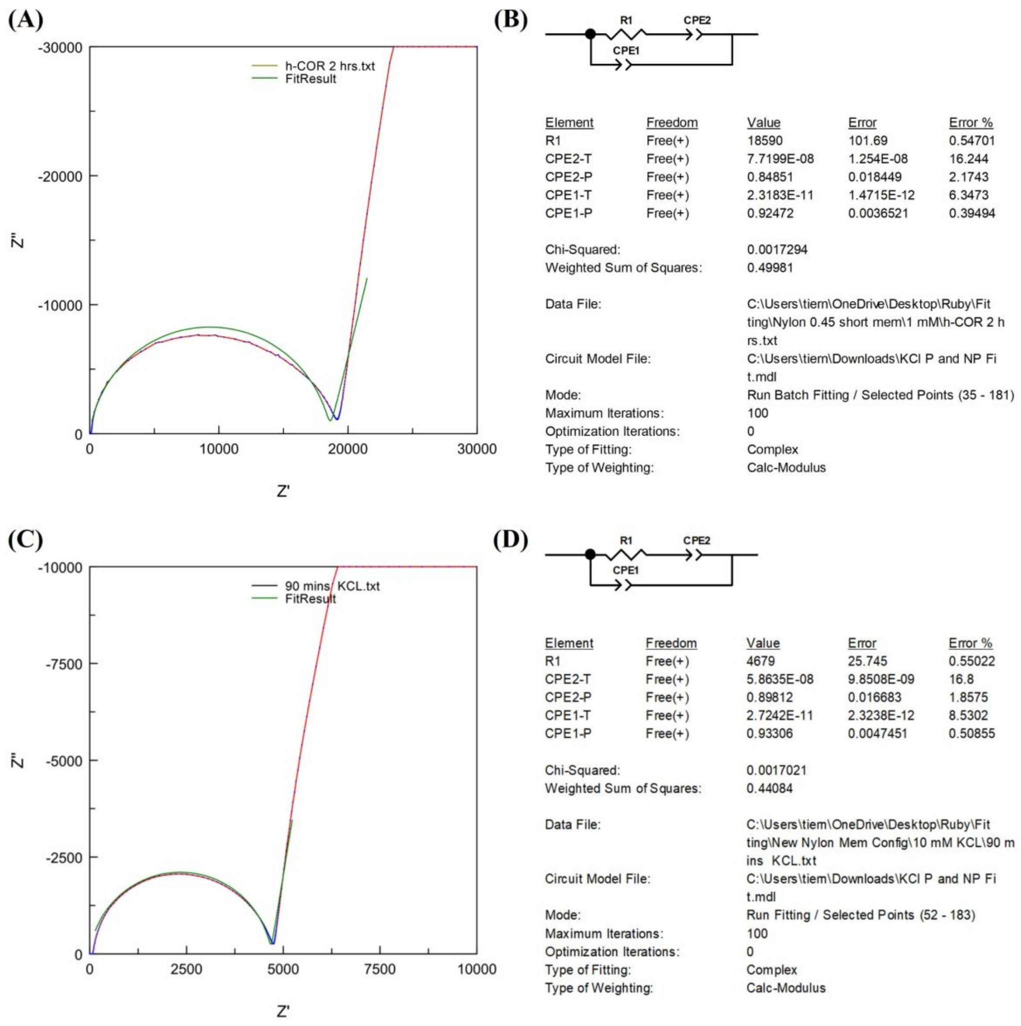

Figure S4: The Z-View fitting results: (A) Raw vs the fitted results for the 1 mM KCl, (B) The circuit used with the obtained parameters for the 1 mM KCl, (C) Raw vs the fitted results for the 10 mM KCl, (D) The circuit used with the obtained parameters for the 10 mM KCl.

The fitting results are shown in **Figure S4. Figure S4A** shows the fitting results for the 1 mM KCl at the end of 2 hrs. The fit and the raw data closely overlap, indicating a good fit, with an error of 0.54% **(Figure S4B).** The no-flow for the 1 mM KCl is shown in **Figures (S5C & S5D). Figure S4C** shows the fitting results for the 10 mM KCl using the parameters shown in **Figure S4D.**

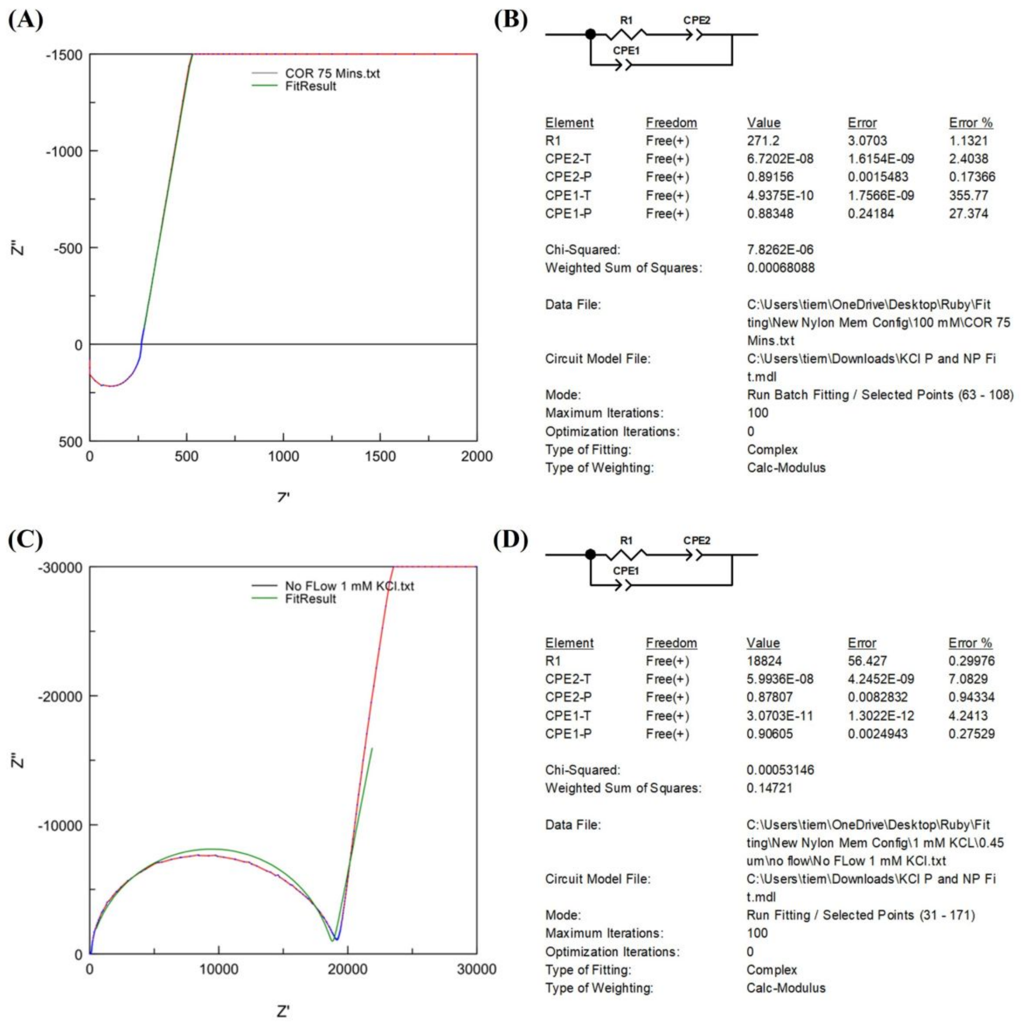

Figure S5: The Z-View fitting results: (A) Raw vs the fitted results for the 100 mM KCl, (B) The circuit used with the obtained parameters for the 100 mM KCl, (C) Raw vs the fitted results for the no-flow 1 mM KCl, (D) The circuit used with the obtained parameters for the no-flow 1 mM KCl.

The fitting results for 100 mM are shown in Figure S5A and Figure S5B. The fit and the raw data closely overlap, indicating a good fit, with an error of 1.13% (Figure S5B).

Section S4. FTIR spectra analysis of the membrane pre- and post-PS bead exposure

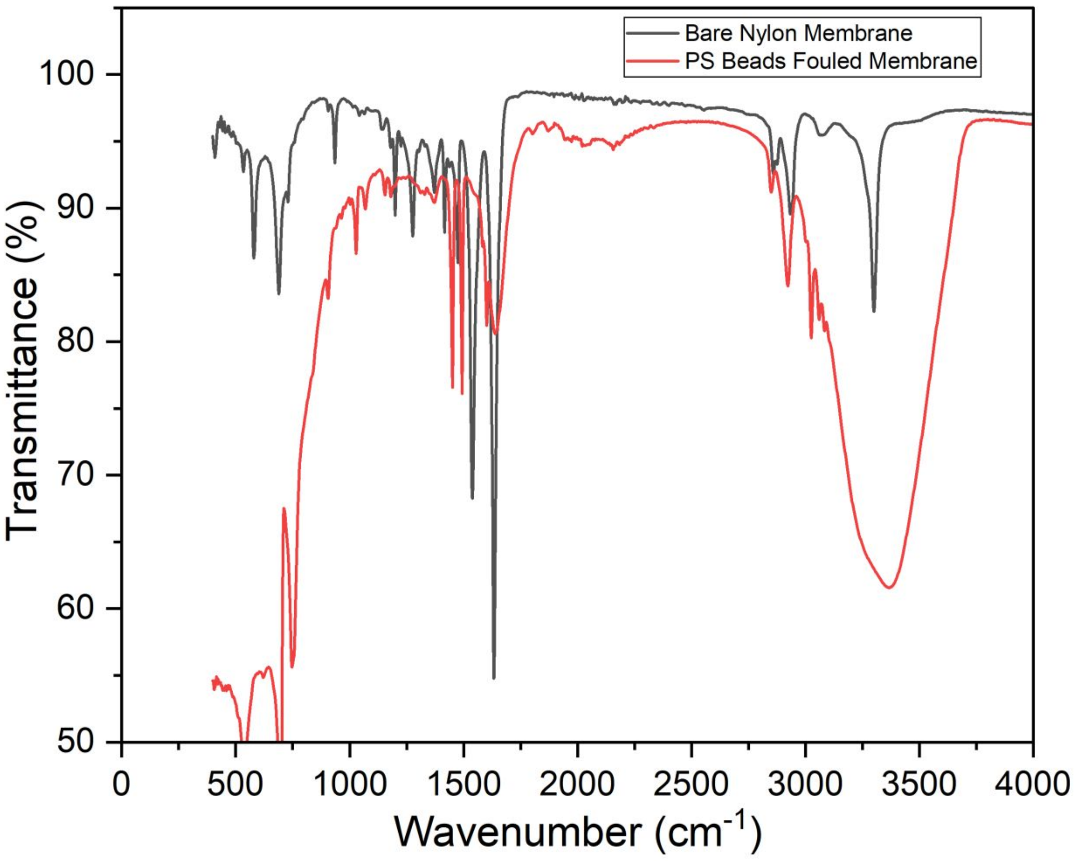

Figure S6: The FTIR spectra of the bare nylon membrane (black) and the PS bead-fouled membrane (red).

The FTIR spectra of the bare nylon membrane and the membrane fouled by PS beads provide clear evidence of surface modification after PS deposition, as shown in **Figure S6.** The spectrum of the bare nylon membrane (black curve) shows characteristic absorption bands at ~3300 cm^{-1} (N–H stretching), ~1634 cm^{-1} (amide I, C=O stretching), and ~1535 cm^{-1} (amide II, N–H bending coupled with C–N stretching), which are typical features of the polyamide backbone.[2-4] These peaks confirm the chemical integrity of the pristine nylon membrane.[4] After the introduction of PS beads (red curve), notable spectral differences are observed. The amide I and II bands decrease in intensity, indicating that the nylon surface is partially masked by the PS

layer, which limits IR penetration into the underlying membrane. Additionally, new absorption features appear around 3025–3080 cm^{-1} and 760–700 cm^{-1}, corresponding to aromatic C–H stretching and ring deformation vibrations of polystyrene. The reduction in amide peak intensity, together with the emergence of PS-related aromatic bands, confirms successful deposition of the PS layer on the membrane surface.

Section S5: Cross-sectional SEM analysis of membrane fouling

To determine whether PS beads enter the membrane pores or remain confined to the surface, cross-sectional SEM imaging was performed on membranes fouled at 10^5 particles/mL **(Figure S7)**. The images show that beads predominantly accumulate at the membrane surface and at the pore entrance. The internal membrane structure does not exhibit embedded particles within the pore channels, indicating that fouling occurs primarily via surface deposition and reduced pore accessibility rather than internal pore filling. These observations are consistent with the membrane's nominal pore rating and support the interpretation of the impedance results as arising from surface-associated blockage and restricted ionic transport pathways.

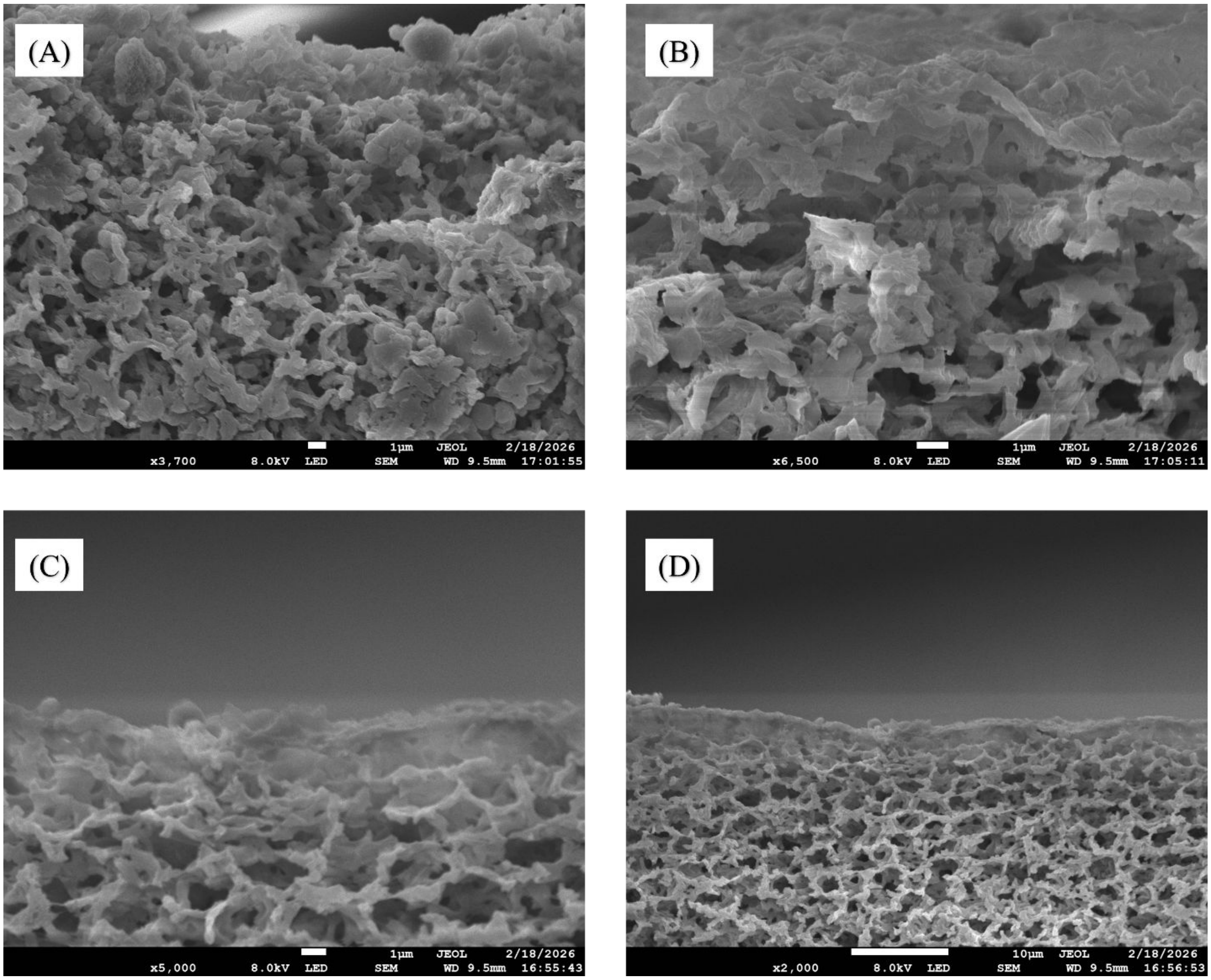

Figure S7 (A-D): Cross-sectional SEM image of the fouled membrane (10^5 particles/mL), confirming that PS beads predominantly accumulate at the membrane surface and pore entrances, with no evidence of deep penetration into the membrane pores.

Section S6. Effect of particle concentration on fouling progression in the IM3 cassette

Figure S8A presents the Nyquist plots obtained when polystyrene beads (10^5 particles/mL) were introduced into the IM3 cassette under continuous flow to foul the nylon membrane. The baseline spectrum in 10 mM KCl (black curve), recorded after the system had stabilized, represents the clean membrane condition. Upon introduction of the beads, the Nyquist plots exhibited a

progressive rightward shift over time, reflected by an increase in the semicircle diameter. These changes indicate a gradual rise in interfacial resistance as bead deposition blocked membrane pores and restricted ionic transport across the membrane. This rightward shift suggests a steady increase in the charge transfer resistance (R_{ct}) and overall system impedance. The mechanism is attributed to bead accumulation on and within the membrane pores, which blocks ionic transport pathways and increases the effective resistance across the electrode–membrane–electrolyte interface. At early time points (1–5 min), the spectra show moderate changes, suggesting initial pore blockage. Beyond ~8 min, the impedance increase becomes more pronounced, consistent with the formation of a bead layer (cake) on the membrane surface. At later time points (34–55 min), the Nyquist plots exhibit large semicircles with high resistance values, indicating severe fouling and significant restriction of ion transport.

Figures (S8B & S8C) show the Nyquist plots obtained when lower concentrations of polystyrene beads (10^3 and 10^1 particles/mL, respectively) were introduced into the IM3 cassette under continuous flow. In both cases, the baseline spectrum in 10 mM KCl (black curve), recorded after stabilization, represents the clean membrane condition. **Figure S8B** shows the Nyquist plots obtained when a lower concentration of polystyrene beads (10^3 particles/mL) was introduced into the IM3 cassette under continuous flow to foul the nylon membrane. Upon bead introduction, the Nyquist plots gradually shifted to the right over time, accompanied by an increase in the semicircle diameter. However, the magnitude of these changes was less pronounced than in the 10^5 particles/mL case **(Figure S8A).** The relatively minor shift in impedance response indicates that at this bead loading, fouling occurs more slowly, with fewer pores blocked and less extensive cake formation on the membrane surface. Early time points (1–5 min) show moderate increases in resistance, consistent with initial deposition and partial pore block. At later times (21–55 min), the

spectra continue to shift, suggesting progressive fouling, but the overall increase in R_{ct} remains significantly lower than that observed for the higher particle concentration.

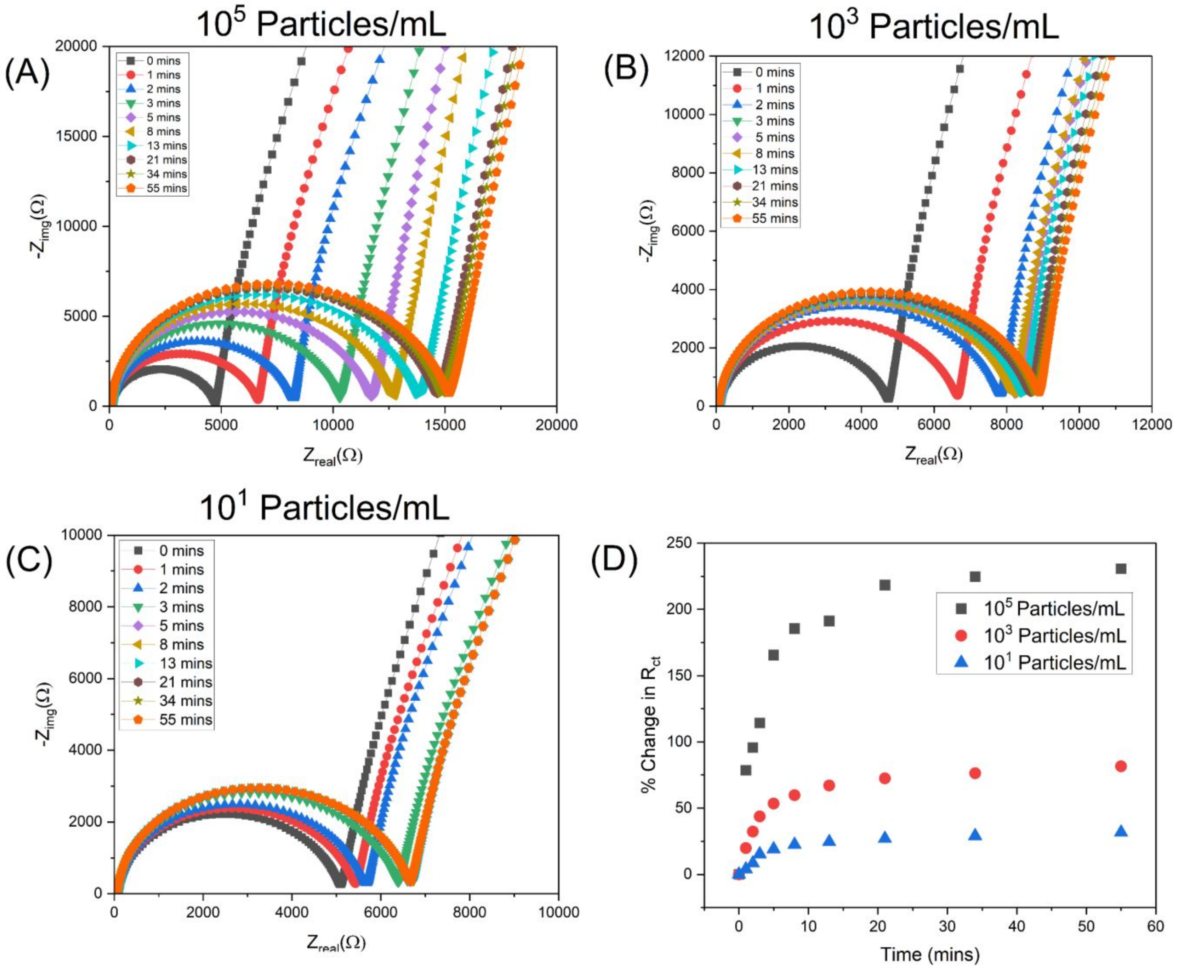

Figure S8: Fouling characterization of the IM3 cassette using 800 nm polystyrene latex beads suspended in 10 mM KCl. (A) Nyquist spectra for 10^5 particles/mL showing rapid impedance increases with time, consistent with severe fouling and pore blockage. (B) Nyquist spectra for 10^3 particles/mL showing moderate, progressive increases indicative of partial pore obstruction. (C) Nyquist spectra for 10^1 particles/mL showing only minimal changes, reflecting negligible fouling. (D) Comparison of % change in R_{ct} over time.

In contrast, the 10^1 particle/mL case **(Figure S8C)** exhibited minimal fouling over the 55-minute experimental period. The Nyquist plots displayed slight rightward shifts, accompanied by slight increases in semicircle diameter, indicating that ionic resistance rose only marginally with time. These subtle changes suggest that only a small fraction of membrane pores were intermittently obstructed, while most transport pathways remain unobstructed and active. Consequently, ionic transport across the membrane was maintained at near-baseline levels, and the extent of fouling under these conditions was less when compared to the higher particle loadings.

The fouling studies performed with polystyrene beads at varying concentrations (10^5, 10^3, and 10^1 particles/mL) demonstrate the IM3 cassette's ability to capture concentration-dependent fouling dynamics in real time with high sensitivity. At high particle loading (10^5 particles/mL), rapid and severe fouling was observed, with pronounced rightward shifts in Nyquist plots reflecting extensive pore blockage and cake layer formation. At intermediate loading (10^3 particles/mL), fouling occurred more gradually, with moderate increases in impedance consistent with partial pore obstruction and slower surface accumulation. At the lowest loading (10^1 particles/mL), only slight changes in impedance were detected, indicating minimal pore blocking and largely preserved ionic transport. This validates the platform as a powerful tool for studying fouling kinetics and advancing predictive strategies for membrane performance.

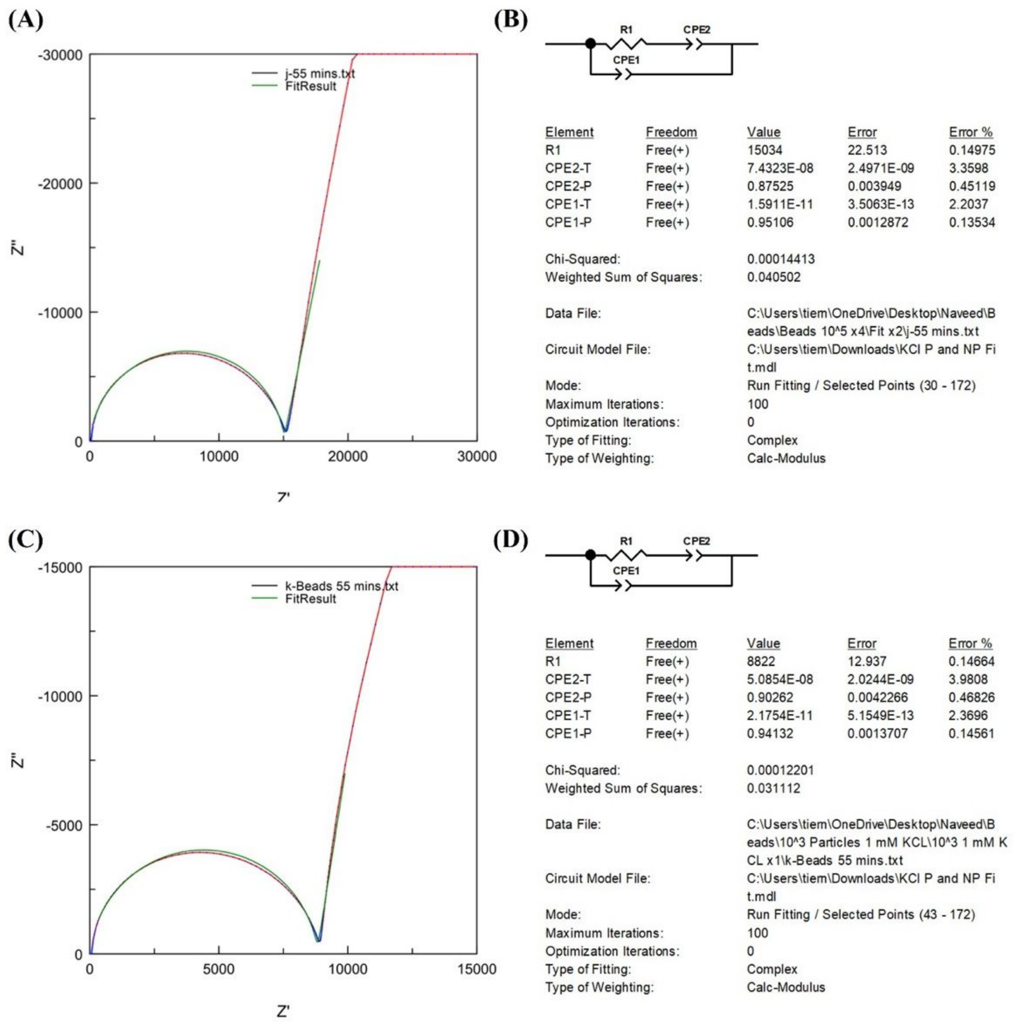

Figure S9: The fitting results for (A) 10^5 particles/mL at 55 minutes, (B) Parameters for fitting of the 10^5 particles/mL, (C) 10^3 particles/mL at 55 minutes, and (D) Parameters for fitting of the 10^3 particles/mL.

The fitting results for the 10^5 and 10^3 particles/mL are shown in **Figure S9**. **Figures (S9A & S9B)** show the fit results for the 10^5 particles/mL at 55 minutes. **Figures (S9C & S9D)** show the fit results for the 10^3 particles/mL at 55 minutes. The fitting results for the 10^1 particles/mL are shown in **Figures (S10A & S10B)**.

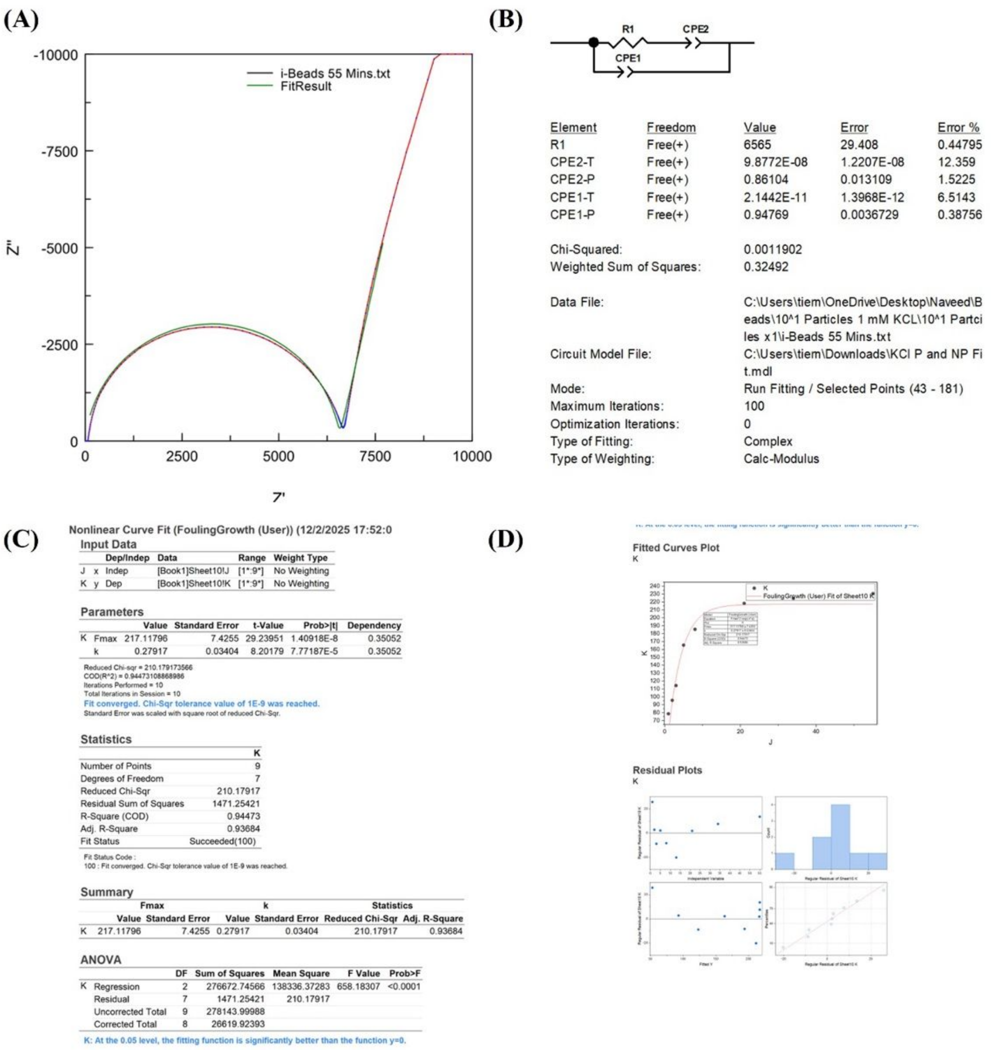

Figure S10: The Fitting results for (A) 10^1 Particles/mL at 55 minutes, (B) Parameters for fitting of the 10^1 Particles/mL, (C) The non-linear fit data that describes the F_{max} and k for the 10^5 particles/mL, (D) The fit and the residual plots for the 10^5 particles/mL.

To model the time-dependent fouling using **Equation 2** as an exponential growth function, as described in the main manuscript. The F_{max} and k, as described in **Equation 3,** are predicted by using the non-linear curve-fitting tool in the Origin Pro software. The F_{max} and k for the 10^5 particles/mL are shown in **Figure S10C**. The fitted and the residual plots for the 10^5 particles/mL are shown in **Figure S10D**.

(A)

Nonlinear Curve Fit (FoulingGrowth (User)) (10/6/2025 12:10:37)

Parameters

		Value	Standard Error	t-Value	Prob>|t|	Dependency
%Rct	Fmax	74.53259	2.12852	35.01617	4.84061E-10	0.35904
	k	0.261	0.02611	9.99675	8.50881E-6	0.35904

Reduced Chi-sqr = 16.6596817876
COD(R^2) = 0.97900898063088
Iterations Performed = 7
Total Iterations in Session = 7
Fit converged. Chi-Sqr tolerance value of 1E-9 was reached.
Standard Error was scaled with square root of reduced Chi-Sqr.

Statistics

	%Rct
Number of Points	10
Degrees of Freedom	8
Reduced Chi-Sqr	16.65968
Residual Sum of Squares	133.27745
R-Square (COD)	0.97901
Adj. R-Square	0.97639
Fit Status	Succeeded(100)

Fit Status Code :
100 : Fit converged. Chi-Sqr tolerance value of 1E-9 was reached.

Summary

	Fmax		k		Statistics	
	Value	Standard Error	Value	Standard Error	Reduced Chi-Sqr	Adj. R-Square
%Rct	74.53259	2.12852	0.261	0.02611	16.65968	0.97639

ANOVA

		DF	Sum of Squares	Mean Square	F Value	Prob>F
%Rct	Regression	2	31868.66133	15934.33066	956.46069	<0.0001
	Residual	8	133.27745	16.65968		
	Uncorrected Total	10	32001.93878			
	Corrected Total	9	6349.2607			

(B)

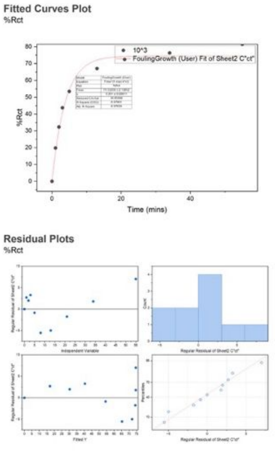

(C)

Nonlinear Curve Fit (FoulingGrowth (User)) (10/6/2025 12:11:42)

Parameters

		Value	Standard Error	t-Value	Prob>|t|	Dependency
%Rct	Fmax	29.00192	0.98218	29.52817	1.87519E-9	0.40091
	k	0.19497	0.02148	9.07491	1.7435E-5	0.40091

Reduced Chi-sqr = 2.97943002475
COD(R^2) = 0.97783658454682
Iterations Performed = 6
Total Iterations in Session = 6
Fit converged. Chi-Sqr tolerance value of 1E-9 was reached.
Standard Error was scaled with square root of reduced Chi-Sqr.

Statistics

	%Rct
Number of Points	10
Degrees of Freedom	8
Reduced Chi-Sqr	2.97943
Residual Sum of Squares	23.83544
R-Square (COD)	0.97784
Adj. R-Square	0.97507
Fit Status	Succeeded(100)

Fit Status Code :
100 : Fit converged. Chi-Sqr tolerance value of 1E-9 was reachec

Summary

	Fmax		k		Statistics	
	Value	Standard Error	Value	Standard Error	Reduced Chi-Sqr	Adj. R-Square
%Rct	29.00192	0.98218	0.19497	0.02148	2.97943	0.97507

ANOVA

		DF	Sum of Squares	Mean Square	F Value	Prob>F
%Rct	Regression	2	4336.23185	2168.11593	727.69486	<0.0001
	Residual	8	23.83544	2.97943		
	Uncorrected Total	10	4360.06729			
	Corrected Total	9	1075.44075			

ct: At the 0.05 level, the fitting function is significantly better than the function y=0.

(D)

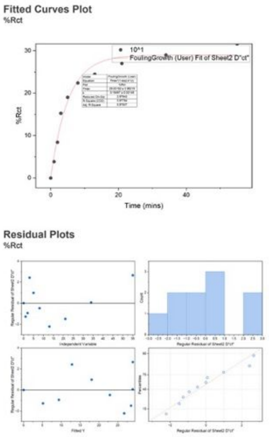

Figure S11: (A) The non-linear fit data that describes the F_{max} and k for the 10^3 particles/mL, (B) the fit and the residual plots for the 10^3 particles/mL, (C) The non-linear fit data that describes the F_{max} and k for the 10^1 particles/mL, and (D) The fit and the residual plots for the 10^1 particles/mL.

The F_{max} and k for the 10^3 particles/mL are shown in **Figure S11A**. The fitted and the residual plots for the 10^3 particles/mL are shown in **Figure S11B**. For the 10^1 particles/mL, the F_{max} and k are shown in **Figure S11C,** and the fitted and residual plots are shown in **Figure S11D**.

The m and n parameters, as described in **Equation 4**, are determined using the linear fitting tool of Origin Pro. The fitted parameters are defined in **Figures (S12A & S12C),** and the corresponding fitted and residual plots are shown in **Figures (S12B & S12D).** Using both linear and non-linear fitting tools yields the empirical model for fouling shown in **Equation 5.**

(A)

Linear Fit (12/4/2025 12:36:50

Parameters

		Value	Standard Error	t-Value	Prob>|t|
E	Intercept	1.23479	0.02683	46.01513	0.01383
	Slope	0.21857	0.00786	27.82062	0.02287

Slope is significantly different from zero (See ANOVA Table).
Standard Error was scaled with square root of reduced Chi-Sqr.

Statistics

	E
Number of Points	3
Degrees of Freedom	1
Residual Sum of Squares	4.93772E-4
Pearson's r	0.99935
R-Square (COD)	0.99871
Adj. R-Square	0.99742

Summary

	Intercept		Slope		Statistics
	Value	Standard Error	Value	Standard Error	Adj. R-Square
E	1.23479	0.02683	0.21857	0.00786	0.99742

ANOVA

		DF	Sum of Squares	Mean Square	F Value	Prob>F
E	Model	1	0.38217	0.38217	773.98684	0.02287
	Error	1	4.93772E-4	4.93772E-4		
	Total	2	0.38267			

At the 0.05 level, the slope is significantly different from zero.

(B)

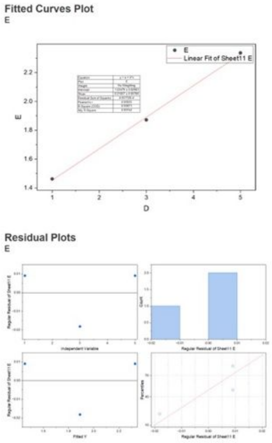

(C)

Linear Fit (12/4/2025 12:40:17

Parameters

		Value	Standard Error	t-Value	Prob>|t|
F	Intercept	-0.73276	0.04803	-15.25593	0.04167
	Slope	0.03897	0.01406	2.77159	0.22044

Slope is NOT significantly different from zero (See ANOVA Table).
Standard Error was scaled with square root of reduced Chi-Sqr.

Statistics

	F
Number of Points	3
Degrees of Freedom	1
Residual Sum of Squares	0.00158
Pearson's r	0.94065
R-Square (COD)	0.88482
Adj. R-Square	0.76963

Summary

	Intercept		Slope		Statistics
	Value	Standard Error	Value	Standard Error	Adj. R-Square
F	-0.73276	0.04803	0.03897	0.01406	0.76963

ANOVA

		DF	Sum of Squares	Mean Square	F Value	Prob>F
F	Model	1	0.01215	0.01215	7.68172	0.22044
	Error	1	0.00158	0.00158		
	Total	2	0.01373			

At the 0.05 level, the slope is NOT significantly different from zero.

(D)

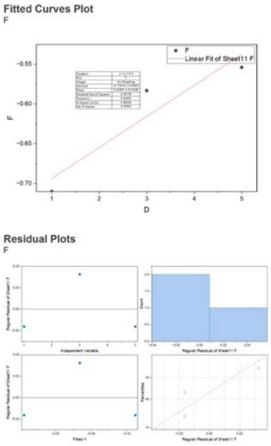

Figure S12: (A) The linear fit data that describes the m and n for the final predicted model, (B) The fit and the residual plots for m and n parameters, (C) The linear fit data that describes the m and n for the final predicted model, (B) The fit and the residual plots for m and n parameters.

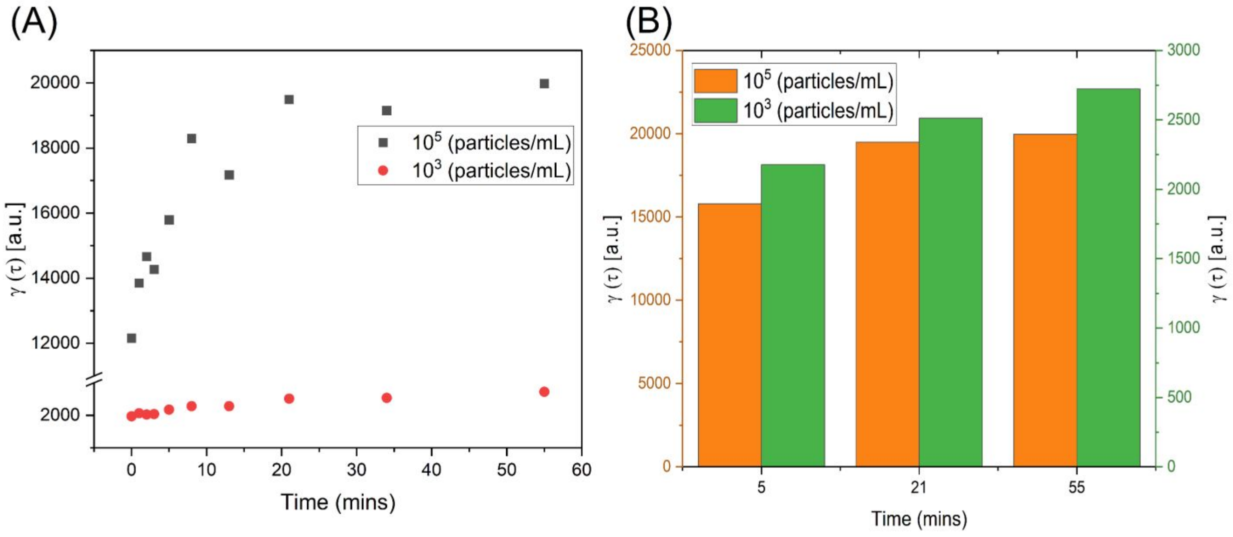

Figure S13: Quantitative evolution of the dominant DRT relaxation peak at ~10^{-2} s during fouling. (A) Time-dependent variation of the peak amplitude extracted from the DRT spectra for 10^5 and 10^3 particles/mL under crossflow conditions. (B) Representative comparison of peak amplitudes at 5, 21, and 55 min for both concentrations. The progressive increase in peak magnitude reflects increasing surface coverage and pore-mouth obstruction with time.

To further clarify the evolution of the dominant peak in DRT analysis, the peak amplitude at ~10^{-2} s was extracted from each DRT spectrum and tracked over time. The extracted values confirm a monotonic increase in peak magnitude during fouling at both particle concentrations, with substantially higher amplitudes at 10^5 particles/mL than at 10^3 particles/mL, as shown in **Figure S13**. This behavior is consistent with progressive surface coverage and reduced ionic accessibility across the membrane-electrode system. While a slight shift in peak position toward longer relaxation times is observed at the highest concentration, no additional peaks emerge, indicating that fouling modulates the magnitude and characteristic timescale of a single dominant interfacial process rather than introducing new relaxation mechanisms.

Section S7. Dose and flux calculations

The membrane dimensions are W = 0.094 in and L = 0.50 in, so the area, A, is

$$A = W \times L = 0.047 \text{ in}^2 = 0.303 \text{ cm}^2 \quad \text{(S1)}$$

For a suspension with concentration C (particles/mL) delivered at volumetric flow Q (mL/min), the areal particle flux to the membrane is:

$$J = \frac{CQ}{A} \text{ (Particles. cm}^{-2}\text{. min}^{-1}\text{)} \quad \text{(S2)}$$

For Q = 3 μL. min^{-1} = 0.003 mL min^{-1}

J = 0.0099*C

Cumulative Particle Dose is:

$$N = \int_0^t J\, dt = Jt = \frac{CQt}{A} \text{ (particles.cm}^{-2}\text{)} \quad \text{(S3)}$$

References

(1) Elbrecht, D. H.; Long, C. J.; Hickman, J. J. Transepithelial/endothelial Electrical Resistance (TEER) theory and applications for microfluidic body-on-a-chip devices. *Journal of Rare Diseases Research & Treatment* **2016**, *1* (3). DOI: https://doi.org/10.29245/2572-9411/2016/3.1026.

(2) Dazzi, A.; Prater, C. B. AFM-IR: Technology and applications in nanoscale infrared spectroscopy and chemical imaging. *Chemical reviews* **2017**, *117* (7), 5146-5173. DOI: https://doi.org/10.1021/acs.chemrev.6b00448.

(3) Tanga, C. Y.; Kwon, Y.; Leckie, J. Effect of membrane chemistry and coating layer on physiochemical properties of thin film composite polyamide RO and NF membranes I. FTIR and XPS characterization of polyamide and coating layer chemistry. *Desalination* **2009**, *242*, 149-167. DOI: https://doi.org/10.1016/j.desal.2008.04.003.

(4) Fazullin, D. D.; Mavrin, G. V.; Sokolov, M. P.; Shaikhiev, I. G. Infrared spectroscopic studies of the PTFE and nylon membranes modified polyaniline. *Modern Applied Science* **2015**, *9* (1), 242. DOI: 10.5539/mas.v9n1p242
